# Strategy for the analysis of lignocellulosic biomass to select a viable transformation route in the Colombian context

**DOI:** 10.1007/s11356-024-32975-x

**Published:** 2024-05-02

**Authors:** Sara Piedrahita-Rodríguez, Andrés-Felipe Alzate-Ramírez, Stéphanie Baumberger, Laurent Cézard, Mariana Ortiz-Sánchez, Diego Alexander Escobar García, Ana María Zetty Arenas, Konstantinos Moustakas, Carlos Ariel Cardona Alzate

**Affiliations:** 1 Departamento de Ingeniería Química, Instituto de Biotecnología y Agroindustria, Universidad Nacional de Colombia, 170003 Manizales, Caldas Colombia; 2 Institut Jean-Pierre Bourgin (IJPB), INRAE, AgroParisTech, Université Paris-Saclay, 78000 Versailles, France; 3 Sede Manizales, Facultad de Ingeniería y Arquitectura, Departamento de Ingeniería Civil, Grupo de Investigación en Movilidad Sostenible (GIMS), Universidad Nacional de Colombia, Campus La Nubia, 170003 Manizales, Caldas Colombia; 4 Unit of Environmental Science & Technology, School of Chemical Engineering, National Technical University of Athens, Athens, Greece

**Keywords:** Agroindustrial residues, Biorefineries, Criteria model, Geographical study, Valorization alternatives, Process sustainability

## Abstract

Lignocellulosic biomass is widely available in the world. However, a consensus has yet to be established to evaluate the biomass valorization alternatives. The chemical composition is the primary technical limitation in selecting a transformation route to obtain value-added products. In this paper, the bagasse from non-centrifuged sugar (NCS) production and *Pinus patula* (PP) wood chips were analyzed in terms of complete chemical composition to establish their potential for selecting the transformation routes. A strategy to select the best route based on the chemical composition was applied and a feedstock criteria model was proposed. Schemes were obtained and compared using a bioprocess selection strategy proposed in previous works. As a result, the preliminary biorefinery schemes were finally defined. The assessment of schemes derived from the outlined strategy included technical, economic, environmental, and social analyses. The environmental evaluation was complemented with a geolocation assessment, revealing a 0.75-ton CO_2_-eq/yr contribution to the carbon footprint for local distribution. The sustainability index for the PP biorefinery and the bagasse from NCS production was analyzed, resulting in indices of 44.8 and 60.9, respectively. These values were primarily derived from the economic and environmental analyses of both processes.

**Supplementary Information:**

The online version contains supplementary material available at 10.1007/s11356-024-32975-x.

## Introduction

Lignocellulosic biomass is considered the most abundant renewable natural resource on Earth (de Bhowmick et al. 2018). It has been used for multiple purposes, mainly as a fuel and heat energy source, to produce wooden implements in the paper industry (Wei et al. 2017). Lignocellulosic biomass derives from plant cell walls, and recent studies have focused on its potential for producing value-added products (Ashokkumar et al. 2022). For example, the conversion of biomass, as part of urban waste upcycling, involves the utilization of a recyclable solid acid catalyst for the transformation of levulinic acid platform molecules into diverse energy vectors, including biogas, ethanol, syngas, and others. The established understanding underscores that processing raw renewable materials mitigates greenhouse gas (GHG) emissions and diminishes reliance on fossil resources. The intricacy of valorizing residual raw materials lies in delineating the optimal transformation route to acquire targeted products. While pertinent, analyses founded on yields or productivity do not singularly constitute the conclusive criteria for process definition. A complete analysis can be proposed with energy, economic, environmental, logistical, and social calculations (Tan 2020). Additionally, these analyses are mutually supportive, and decision-making can be much more complex and limited by context. Different aspects must be analyzed using a biorefinery approach to determine a suitable transformation route (according to the raw material) (Solarte-Toro et al. 2023). Initially, the technical aspects play a significant role since they restrict the minimum conditions that must be considered to generate the products by a selected transformation route.

However, considerations in terms of sustainability validate the possibility of applying a proposed process in any context. Economic, environmental, and social limitations can generate different results in the selection. The purpose is to find a balance between these pillars, where the results can differ if validated under different scales and country contexts, and even consider logistical aspects, such as the processing plant location and targeted market. Considering the elements of sustainability and the context, a clear strategy is required to address this challenge. Significant advances have been made from different perspectives (Table 1). Cardona-Alzate et al. (2019) established that bioproducts or bioprocesses can be identified from the conceptual design stage, according to knowledge-based design. This approach involves analyzing raw material components and establishing primary bioproducts, employing hierarchy, sequencing, and integration concepts. Palmeros Parada et al. (2020) described a midstream modulation for designing and evaluating processes, specifically for biorefineries design. The aim is to articulate stakeholders’ objectives throughout the design process. Nevertheless, these biomass transformation proposals do not ensure the optimal alternative. Ortiz-Sánchez and Cardona Alzate (2022) recognized the necessity for a strategy encompassing more critical aspects to thoroughly analyze and select the best transformation route. This strategy facilitates defining and evaluating optimal transformation routes aligned with a sustainability objective. Despite these advances, it is necessary to complement and validate the schemes starting from the lignocellulosic biomass composition (cellulose, hemicellulose, lignin, extractives, and ash), which is the main limiting factor in the technical definition of recovery processes. Knowing the biomass composition enables identifying the potential of transformation and proposing valorization processes for each biomass fraction to understand its real possibilities (Sharma et al. 2017; Den et al. 2018; Machineni 2019). The idea is to identify the boundaries where processes are feasible and integrate them into more complex systems, such as biorefineries.

**Table 1:** Characteristics and limitations of current studies regarding the design and selection of transformation routes from biomass

Reference	Main characteristics	Limitations
Cardona-Alzate et al. (2019)	Conceptual design stage, to define bioproducts and bioprocesses, applying the concepts of hierarchy, sequencing, and integration	Not all the schemes proposed are viable. No quantitative information is used
Palmeros Parada et al. (2020)	Midstream modulation for the design and evaluation of processes	No quantitative information is used. It is a bit subjective
Ortiz-Sánchez and Cardona Alzate (2022)	Strategy for the selection of bioprocess according to bioproducts portfolio. It uses some techno-economic and environmental metrics	Some key aspects are no considered, such as geographical information, a complete characterization of the raw material and social aspects

Colombia has a vast biomass potential where agroindustrial and forestry waste are the most common biomass sources available (Gutiérrez-Mosquera et al. 2018). The search for valorizing alternatives for this waste has become a key aspect of the bioeconomy establishment. The purpose is to assess value chains, identify the residue, and return them to the same system or another value chain, achieve integration of chain links, and guarantee at least economical, technical, and environmental pre-feasibility. An example of representative value chains in Colombia is non-centrifuged sugarcane and pine. The crop of sugarcane (*Saccharum officinarum*) from non-centrifuged sugar production (NCS) is important and representative at the cultural, economic, and social levels (Silva Lora et al. 2016). In Colombia and India, this value chain allows the subsistence of many families. Nevertheless, the most critical residue in the value chain is generated during sugarcane harvesting and processing. The bagasse from NCS production is a by-product from panela (brown cake containing mainly sucrose) production, specifically in the sugarcane juice extraction stage. It represents more than 30% wt (wet basis) of the waste generated in the value chain, which is worth analyzing (Ramírez Durán et al. 2014). The energy generation needed in the panela production process is the current use of this bagasse from NCS production. However, it has high moisture and low calorific value (Gutiérrez-Mosquera et al. 2018). Thus, the bagasse is usually mixed with wood and other residues to fulfill the total process energy requirements. In addition, most of the NCS producers work on a small scale, indicating their low technology (Vanegas Salazar and Mario 2016). On the other hand, wooden plantations, such as *Pinus patula* (pine), represent an important source of lignocellulosic materials and are one of the most promising species in terms of reforestation in Colombia (mainly in the central zone of the country and paramo areas), due to their ideal geographic, climatic, and topographic conditions (García et al. 2017). *Pinus patula* wood chip (PP) is attractive due to its relatively high yields compared to other timber species (35 m^3^/ha year) (García et al. 2017). The industrialization of this raw material generates significant amounts of residues (wood chips and sawdust represent more than 65%). The PP is currently disposed in the field or taken to landfills and burned (Moncada et al. 2016b).

Therefore, this paper proposed a mode for selecting the best transformation route for PP and NCS bagasse raw materials based on their chemical composition. First, a complete characterization and a geographical analysis were conducted to select the biorefinery schemes based on the literature. Second, a criteria model was proposed for the selection. Third, a superstructure of possible biorefineries was created, and the scheme was compared to other bioprocess selection strategies proposed by Ortiz-Sánchez and Cardona Alzate (2022). The aim was to obtain a model capable of proposing biorefinery systems based on the composition of any raw material and context. Finally, the biorefinery scheme was defined based on the superstructure and evaluated in terms of sustainability.

## Materials and methods

### Global scope

Initially, the bagasse from NCS production and PP were analyzed in terms of composition to determine the best preliminary lignocellulosic biomass transformation schemes. A complete analysis was performed, including lignocellulosic chemical composition (with characterization of phenolic compounds) and total and volatile solids analysis. Subsequently, a context study based on geographical information was performed to understand the relevance of life cycle and value chain analysis. A criteria model was proposed based on the complete information on the raw materials composition. A well-established bioprocess selection strategy developed by Ortiz-Sánchez and Cardona Alzate (2022) was applied to the raw materials. Then, the schemes were compared by identifying the differences and similarities. The scope of both studies was to reach the NCS bagasse and PP valorization proposals. Finally, one biorefinery scheme was selected and evaluated in terms of sustainability pillars for each raw material. The comparison of the schemes shows the relevance of the composition in the definition of sustainable biorefineries. Therefore, the overall methodology of this paper was divided into four sections, initially involving the experimental part (complete feedstock composition and geographical analysis), study 1 (criteria model), study 2 (bioprocess selection strategy), and evaluation of a biorefinery scheme.

### Experimental part

#### Raw material

The raw materials correspond to lignocellulosic residues obtained in the transformer link of the value chains in Colombia. The NCS bagasse was kindly provided from a sugarcane mill in the central region of Colombia (5°24′47″N 74°59′34″W). On the other hand, PP was obtained from a board and chipboard manufacturing company located in the central mountains of Colombia (5°03′58″N 75°29′05″W). Each raw material was air-dried and grounded to particle sizes of approximately 4 mm, which is crucial to characterize according to international standards. Besides, the NCS bagasse and PP were stored and protected from direct light and moisture sources.

#### Chemical analysis

The bagasse from NCS production and PP were analyzed in terms of their chemical composition, following the international standards: for moisture determination, ASTM E871-82; extractives in water and ethanol, NREL/TP-510–42619; ash, NREL/TP-510–42622; total and volatile solids (TS &VS) described by NREL/TP-510–42621, and cellulose and hemicellulose, ASTM D1104. The determination of lignin was performed according to the Klason method (NREL/TP-510–42618) and the lignin monomers were characterized through the thioacidolysis process (Majira et al. 2019). All tests were performed in triplicate, and the methodology is described in Supplementary Material 1 (SM1).

#### Geographical analysis

**Fig. 1:**
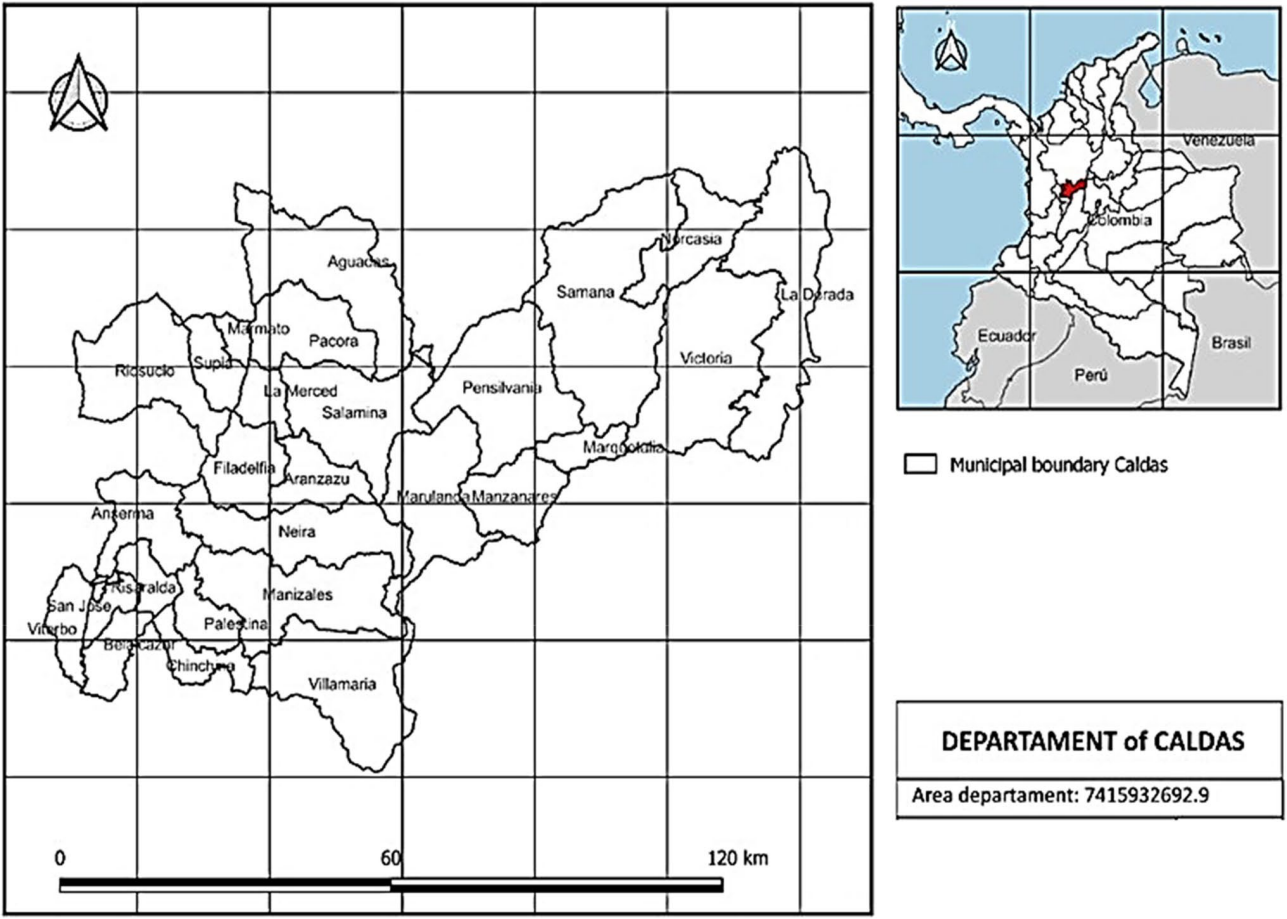
Caldas region considered in this work

The raw materials analyzed in this paper were contextualized to the region of the department of Caldas, Colombia (Fig. 1). This location has the particularity of being a coffee-growing region, with a variety of other crops, such as sugarcane and plantain. For the geographical analysis, the information on sugarcane from the NCS production and forest crops was considered to evaluate the synergy between these crops and to understand the need to look for alternatives for using their residues. The cultivation of sugarcane from the NCS production in Caldas presents low technology. Its energy efficiency is low due to the sugarcane mills operating by burning wet bagasse supplemented with wood from nearby forests and, sometimes, charcoal. However, the burning efficiency is much higher when the bagasse is dried. Therefore, the climate of the region, defined by temperatures and rainfall, was included in the study. This geographic analysis provides a context of the raw materials and shows their importance for decision-making regarding implementing new technologies and selecting the transformation routes. In addition, a life cycle analysis to understand the impact of using the proposed raw materials to produce several products was included. This analysis will be complemented with a final discussion about the criteria model and the connection between the biorefinery schemes and the region maps.

##### Geospatial data acquisition

The first step to performing a geographical analysis is acquiring geospatial data, which was selected based on the conducted analysis, in terms of the raw materials (sugarcane from NCS production and pine forest crop). This analysis was based on metrics and environmental factors such as land use, existing vegetation, and water sources. Data collection began with the information provided by the Landsat 8 satellite which includes raster data (pixels) that allows the generation of an image of the study region by overlaying the satellite data bands (Pettorelli et al. 2005; Martinuzzi et al. 2008). The image construction with raster data was performed using QGIS version 3.28.4 geospatial analysis software. Information was collected from government databases stored in vector files (i.e., Shapefile, GeoPackage) represent the region’s characteristics visually. Different strategies were used to visualize better the information in the images, such as establishing different symbology and gradients to differentiate terrain features, scale, transparency, projection adjustments, and a combination of varying vector file layers (Guevara-Ochoa et al. 2018). Furthermore, to guarantee the homogeneity and applicability of the data, an information period of 3 years for the sugarcane and pine crops was considered.

In addition, a soil organic carbon sequestration (SOCseq) map was constructed to determine the contribution to climate change mitigation and its correlation with the other information based on context. The SOCseq map determined the amount of carbon stored in the soil, which could be analyzed under different management scenarios and contributed to climate change mitigation (FAO 2022). With this estimation, sustainable management areas and strategies could be prioritized to reduce greenhouse gases from soil carbon sequestration. The objective was to determine how much organic carbon could be stored in the soil. It also contributed to formulating sustainable management strategies in agricultural and livestock areas (Lefèvre et al. 2017). SOCseq involves the fixation of atmospheric carbon dioxide through plant photosynthesis. Once the plants have completed their cycle, they were deposited in the soil as aerial and underground plant residues (Lefèvre et al. 2017). Finally, the geographic analysis allowed to discuss the impact of using different raw materials in the life cycle and value chain analysis.

##### Geolocation analysis

The information obtained from the geographical analysis will serve as context data necessary for selecting transformation routes. For example, in logistical terms, the location of the transformation route is discussed according to environmental or economic objectives. The geographic analysis included a geolocation analysis regarding distances from the region’s collection centers and distribution routes for this case. This localization was carried out considering constraints superimposed as layers on the maps. The analysis was performed based on the lowest carbon footprint the location generated in terms of transportation of raw materials and products. It was performed with the environmental analysis of the scheme obtained (the “Environmental and geolocation analysis” section). The necessary data were collected from different sources under the Colombian context (Marco Geoestadístico Nacional of Departamento Administrativo Nacional de Estadística, Sistema Nacional de Áreas Protegidas de Colombia, Instituto de Hidrología, Meteorología y Estudios Ambientales, and Open Street Map (OSM)). The limits of the transformation route location were set to 150 km around the centroid of the municipalities of Samaná and Manizales (Piedrahita-Rodríguez et al. 2023a, b). The exclusion-inclusion criterion was implemented to define limiting factors such as urban areas (5 km around), hydrology (50 m around rivers), and National Natural Parks (5 km around protected areas). Finally, 200 random points were placed in the potential zones for the location of the transformation route and evaluated in terms of carbon footprint (CF). The CF was calculated for three different product distribution scenarios: (i) local, (ii) regional, and (iii) national (Piedrahita-Rodríguez et al. 2023a). The local scenario considered the distribution of products obtained from the transformation route to Manizales. The regional scenario considered an equal distribution of products to Manizales and Cali. Finally, the national scenario considered the distribution of products 40% to Medellín (an important city in the region), 40% to Cali, and 20% to Manizales.

### Strategy 1: criteria model proposal

A criteria model was proposed through the characterization of raw materials and its geolocation analysis (Table 2). Initially, the composition of raw materials was located as the first step of the model. The idea was to propose alternatives for valorizing as many raw material fractions as possible. Certain considerations were defined for each fraction, which must be met as a minimum to propose a specific transformation route. In the case of fiber (cellulose, hemicellulose, and lignin), the ratio of cellulose and hemicellulose with lignin was defined (CH/L). This ratio made possible to establish at what point the lignin content could affect the selection of the transformation processes. It was considered since these fractions give rise to platform molecules such as C5 and C6 sugars, essential in biotechnological processes. Based on this data, three levels of CH/L were defined corresponding to values between (i) level 1, 0.60 and 1.60; (ii) level 2, 1.60 and 6.60; and (iii) level 3, 6.60 and 9.60. A literature review was conducted to establish the values of this ratio for different raw materials and the transformation processes that have been studied (generating feasibility cases at the techno-economic level). In this way, it was possible to classify the processes in the defined levels (Table 3). The review and selection of the processes according to the fractions of the raw material are described in Supplementary Material 2 (SM2). Similarly, the other raw material fractions were considered. In the case of lignin monomers, extractives, fats, and protein, a rate was not determined as such, but rather the identification of molecules of interest. If the content of these molecules in the fraction was higher than the limit defined in Table 2, the transformation processes shown in Table 3 could be considered. The processes defined for these raw material fractions were selected according to their flexibility and simplicity; i.e., the first process will correspond to the “simplest” in technical terms or generate lower equipment costs, energy requirements, or negative environmental emissions. The last process will be the most sophisticated technology, considerably more expensive, or include hazardous substances within the process or require independent process lines for disposal. Finally, the Biodegradability Index (BI) was calculated for solids content. The BI was defined as the ratio of VS to TS and established the fraction of biomass that could be anaerobically biodegraded (Lesteur et al. 2009). A value of up to 70% in BI allowed to propose an anaerobic digestion process.

**Table 2:** Compositional criteria model proposed for the first step of the methodological strategy for selecting transformation routes based on lignocellulosic biomass

Raw material composition	Rates and considerations	Lower–upper values	Group
Cellulose Hemicellulose Lignin	$\frac{{\text{Cellulose}}+{\text{Hemicellulose}}}{{\text{Lingin}}}$	Level 1: 0.6–1.6 Level 2: 1.6–6.6 Level 3: 6.6–9.6	Group 1
Monomers of lignin	Phenolic compounds	Content > 50 mg/g	Group 2
Extractives	Phenolic compounds easily recovering	Content > 50 mg/g	Group 3
Volatile and total solids	Biodegradability index (BI)	BI > 70%	Group 4
Protein and fats	Type of molecules	Content > 40%	Group 5

**Table 3:** Processes selected based on the groups defined in compositional criteria model

Group 1	Level 1: Pyrolysis Gasification Kraft	Level 2: Fermentation (TRL > 7) Anaerobic digestion	Level 3: Fermentation (TRL < 7) Catalytic conversion New techniques
Group 2	Pyrolysis (depolymerization) Oxidative depolymerization Microwave-assisted depolymerization Acid/base-catalyzed depolymerization Supercritical fluid extraction		
Group 3	Steam distillation Ultrasound extraction Microwave-assisted extraction Solvent extraction Supercritical fluid extraction		
Group 4	Anaerobic digestion without pretreatment Anerobic digestion with pretreatment		
Group 5	Any process to obtain food products Processes to obtain animal feed		

Finally, a biorefinery structure was proposed once the possible processes were selected. This criteria model aimed to obtain a basic biorefinery scheme that can be applied to using the raw materials under study. Thus, allowing a comparison of the schematic proposals of bioprocesses defined in strategy 1.

### Strategy 2 for selecting the best route

**Fig. 2:**
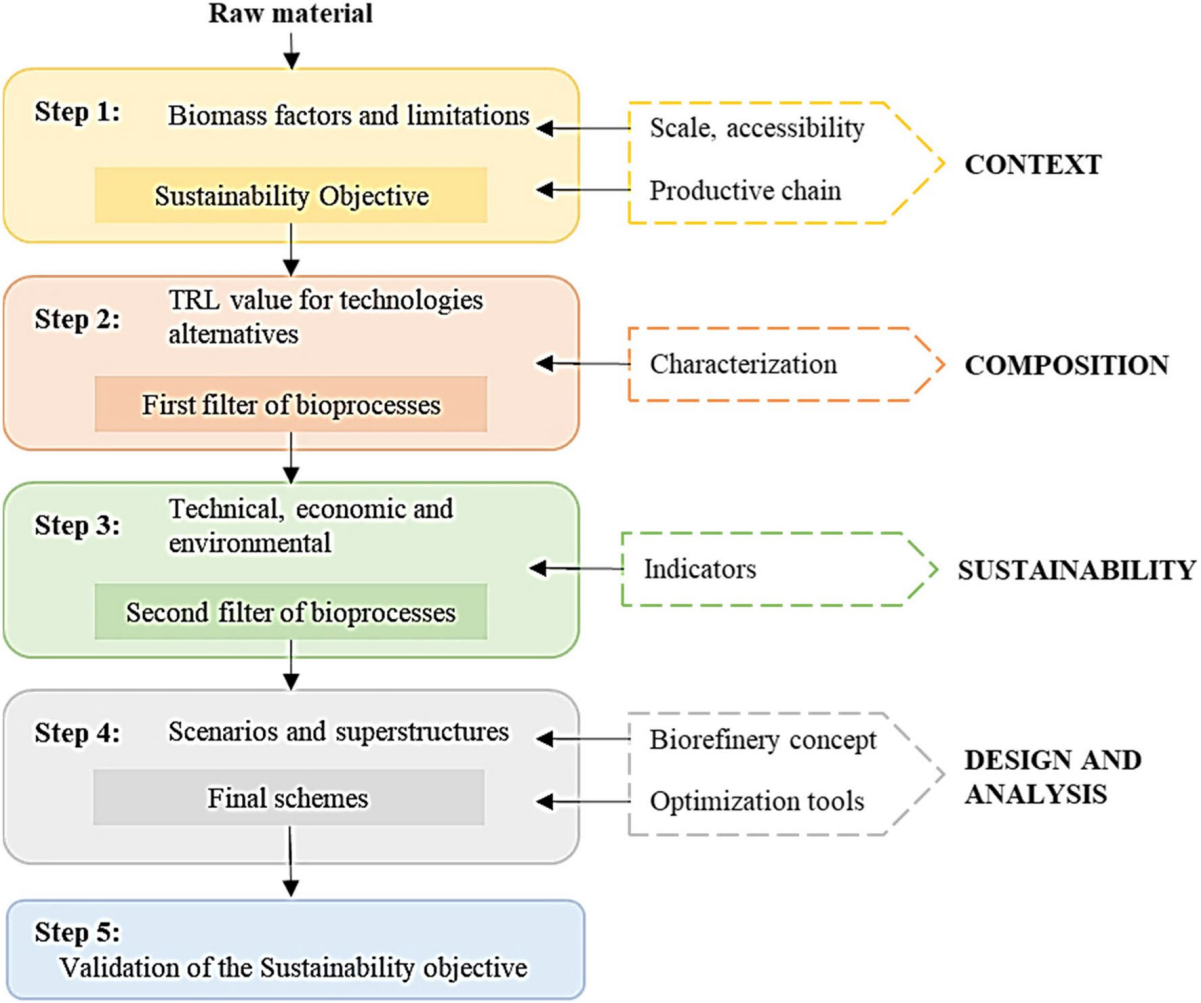
Strategy for bioprocess schemes selection. Adapted from Ortiz-Sánchez and Cardona Alzate 2022

This strategy selected transformation routes from biomass information, based on a portfolio of processes and products defined in the same strategy. The strategy proposed by Ortiz-Sánchez and Cardona Alzate (2022) was applied to select the best transformation route based on the raw material composition. The strategy considered different steps based on rigorous sustainability calculation for the fourth dimension (technical, economic, environmental, and social) to validate the expected results (Fig. 2). Step 1 defined the most relevant constraints for the raw material. For this purpose, the production chain of PP and bagasse from NCS production was generally analyzed. Additionally, the composition found for these biomasses was identified based on experimentally obtained data. The sugarcane from NCS production in Caldas for 2021 was approximately 83.5 Mton (Agronet 2023). The bagasse from NCS production represented about 30% of production, around 25 Mton (Ramírez Durán et al. 2014). On the other hand, about 17 10^7^ kg/year for the case of PP (high scale) productivity was reported, where approximately 2 10^7^ kg/year (small-scale) corresponded to the residues generated in wood harvesting and industrial processing (Ministry of Agriculture and Rural Development 2014).

In 2016, Colombia’s National Planning Department (DNP) reported the innovation ranking for the country’s regions, positioning Caldas as the fifth region on the list (Cámara de Comercio de Manizales por Caldas 2016). These results highlighted the department in terms of growth and development. During the following years, this region has shown progress in economic and social development through the promotion of small businesses and important support to the educational part of the region. Thus, this region has a medium–low technological level, in which innovation is gaining strength through small businesses. The implementation of highly developed (high technology readiness level (TRL)) but flexible technologies was required for this study. Finally, this step allowed to identify the sustainability objective for both cases. Step 2 (Fig. 2) aimed to define bioprocess schemes according to the sustainability objective and the composition and context information of step 1. In this paper, several bioprocesses were proposed for each raw material. This selection of bioprocesses was made based on the analysis of a range of processes proposed in the Ortiz-Sánchez and Cardona Alzate (2022) strategy to identify the products. Furthermore, the technological context and maturity were considered through the TRL for each of these processes. Once the bioprocesses were classified according to their TRL, they were reclassified according to technical, economic, and environmental feasibility (step 3, Fig. 2). Technical indicators were analyzed (mass and energy indicators to guarantee complete use of biomass and lower energy consumption), economic indicators (guaranteeing lower capital consumption and operating costs), and environmental indicators (guaranteeing lower emissions). As suggested by the strategy, the indicators were standardized for each biomass fraction to be considered. Finally, in step 4 (Fig. 2), different scenarios were proposed. These scenarios led to processing approaches such as biorefineries in which the integral use of biomass fractions was considered. However, as they become more complex, the technical, economic, and environmental feasibility may be compromised, and consequently, the transformation schemes must be validated (step 5, Fig. 2). Finally, the scheme obtained was compared to the scheme of strategy 1.

### Evaluation of biorefinery scheme

After analyzing the superstructure of possible biorefineries, one was selected to evaluate in technical, economic, environmental, and social terms (sustainability analysis) and the selection of the transformation route was validated with the criteria model (strategy 1). The selection of the pretreatment stage was carried out experimentally by evaluating three possible pretreatments: (i) dilute sulfuric acid, (ii) alkaline with NaOH, and (iii) dilute trifluoroacetic acid (TFA). The methodology to evaluate the obtained biorefinery is mentioned below.

#### Pretreatment selection

Three schemes were chosen for experimental evaluation to select the best raw material pretreatment. The first scheme consisted of pretreatment with 2% v/v dilute sulfuric acid with a solid–liquid ratio of 1:10, at 120 °C for 1 h (Unrean and Ketsub 2018). For the second pretreatment, sodium hydroxide (1 N), with a solid–liquid ratio of 1:10, was used at 100 °C for 1 h (Maryana et al. 2014). Finally, the third pretreatment used a 2.5 M trifluoroacetic acid (TFA), at a solid–liquid ratio of 1:20, at 120 °C for 2 h (Piedrahita-Rodríguez et al. 2023b). Each sample was conditioned at room temperature and centrifuged for 10 min at 3700 rpm. The supernatant was used for sugars (xylose and glucose), and for inhibitors (furfural and 2,5-hydroxymethylfurfural) quantification by high-performance liquid chromatography (HPLC) (Unrean and Ketsub 2018). Thus, it was possible to complement the process flow diagram to be evaluated by considering each processing stage.

#### Technical analysis

The biorefinery process was simulated in Aspen Plus V.9.0 (Aspen Technologies, Inc., USA). Experimental data served as input for the simulation (characterization and pretreatment results). It was complemented with literature data when necessary. For each analysis, 2.50 ton/h of feedstock (value available in the region initially delimited by the value chain analysis) was taken as the basis for the calculation as a processing flow. The scheme was handled with the non-random two liquids and Hayden-O’Connell (NRTL-HOC) method as a thermodynamic method due to the processing conditions such as temperature and pressure, phases involved, and thermodynamic properties. Finally, the material and energy balances were obtained, allowing indicators to be calculated for the technical analysis. Cellulose, hemicellulose, lignin, enzymes, and microorganism properties were fed to the software. Properties such as formation, combustion, and free energy Gibbs enthalpies were considered (Alengebawy et al. 2022). The methodology for calculating the technical indicators of the proposed biorefinery scheme was defined by GREENSCOPE, developed by the US Environmental Protection Agency (EPA). This methodology allows the determination of indicators for complete processes or stages, making it useful at a comparative level. The technical indicators considered in this work were process mass intensity (PMI) and renewable material index (RMI).

#### Economic evaluation

The economic evaluation of the biorefinery scheme was carried out by evaluating the net present value (NPV), the payback period (PBP), and the turnover index (TR) as comparative metrics. The tax and interest rate values were selected considering the Colombian context (35% and 13%). The project lifetime was 20 years, considering the scale of raw materials processing. The methodology described by Rueda-Duran et al. (2022) was implemented to calculate capital expenditures (CapEx) and operational expenditures (OpEx). Finally, a sensitivity analysis was performed to determine the equilibrium point (scale where profits and expenditures are equal) and the Minimum Processing Scale for Economic Feasibility (MPSEF); i.e., the NPV is zero in the last year of the project lifetime.

#### Environmental and geolocation analysis

The life cycle assessment (LCA) was performed according to ISO 14040 methodology. The main objective of LCA was to determine the carbon footprint of a biorefinery considering the geolocation problem. The mass allocation was used in this analysis, and the functional unit used in the LCA corresponded to 1 kg of the raw material of the biorefinery. The cradle-to-gate perspective was applied attributional analysis (Corona et al. 2018; Rodríguez et al. 2018). The assessment considered the agronomic aspects of the raw materials. All the agrochemicals were considered inputs for each raw material crop, using secondary data and interviews. The transformation link was structured through the conceptual design methodology defined by Cardona et al. (2019) (Moncada et al 2016a). The processing flow data was taken from the Colombian Ministry of Agriculture (Ministerio de Agricultura y Desarrollo Rural, 2023) and information from Caldas producers, specifically in Manizales and Samaná. The inventory of the agronomic stage can be seen in the “Results” section because it includes the PP and the from NCS bagasse processing defined after the criteria model application. The LCA results were analyzed for the three product distribution scenarios in terms of carbon footprint (CF) and water depletion (WD). Besides, the environmental analysis was complemented with geolocation analysis regarding the best location of the biorefinery where the lowest carbon footprint was calculated (the “Geolocation analysis” section).

#### Social assessment

The social analysis was conducted in the Colombian context to obtain the minimum-to-living wage ratio (*M*/*L*) indicator, which is the ratio between the minimum wage paid to an operator and the living wage of a single person in Colombia (Solarte-Toro et al. 2023). The value of *M* was considered in the economic analysis to determine the labor cost. In contrast, the value of *L* is defined as the money needed to have a decent life in a specific context, considering the costs of food, housing, health, and education (WageIndicator 2023). A sensitivity analysis of the social indicator in the economic analysis was carried out without varying the number of operators. In this sense, it was possible to determine the social resilience of the biorefinery to an increase in the minimum wage without affecting the project’s net income. The employment potential the transformer link can generate (biorefinery) was also determined. For this purpose, two types of available jobs were assumed (operators and supervisors) and estimated based on the total working hours of the biorefinery (8400 h/year). Conditions such as the maximum working time per week set at 47 h (law 2102 of 2021 in Colombia), 1 day off per week for operators, and vacation time and holidays stipulated by the Ministry of Labor in Colombia were also considered. The number of operators was increased by one to avoid overlapping shifts during the workday. Additionally, three supervisors were considered per process area, defined by the flow diagram of the biorefinery. This methodology of calculating employment potential was compared with the correlation of Alkhayat and Gerrard (1984).

#### Sustainability index calculation

The biorefinery scenarios were analyzed considering technical, economic, environmental, and social indicators. The global metric SI (sustainability index) was determined to define the sustainability of the processes. This indicator was estimated following the methodology described by Solarte-Toro et al. (2023). Only if the process was economically feasible, the SI can be calculated. The sustainability index was estimated using the following equation:

1 $$SI = {w}_{1}\sum {w}_{n}T{I}_{n}+{w}_{2}\sum {w}_{n}Ec{I}_{n} +{w}_{3}\sum {w}_{n}En{I}_{n}+{w}_{4}\sum {w}_{n}S{I}_{n}$$

where $SI$ is the sustainability index (between 0 and 100%); ${w}_{1}$, ${w}_{2}$, ${w}_{3}$, and ${w}_{4}$ are the global weighting factors used to estimate the influence of each dimension (i.e., technical, economic, environmental, and social); ${w}_{n}$ are the weighting factors of each metric in each dimension; $T{I}_{n}$, $Ec{I}_{n}$, $En{I}_{n}$, and $S{I}_{n}$ are the set of technical, economic, environmental, and social indicators. Each of the indicators has limits defined as the best case and the worst case. These limits allow for locating the calculated values by the biorefinery and determining their impact on calculating the sustainability index. Table 4 shows the best- and worst-case values for each indicator calculated for the four dimensions of sustainability.

**Table 4:** Techno-economic, environmental, and social metrics, and sustainability index (SI) of the biorefineries

Dimension	Indicator	Best case	Worst case
Technical (TI_n_)	Process mass intensity (PMI)	1.00	100
	Renewable material index (RMI)	1.00	0.00
Economic (EcI_n_)	Playback period (PBP)	1.00	20.00
	Turnover ratio (RR)	5.00	0.10
Environmental (EnI_n_)	Carbon footprint (CF)	0.50	20.00
	Water depletion (WD)	1.00	20.00
Social (SI_n_)	Minimum-to-living wage ratio (M/L)	1.00	0.50

## Results and discussion

### Composition of the raw materials, chemical analysis

The characterization of the raw materials evaluated in this study is shown in Table 5. Despite being two different raw materials, their total fiber content is above 50% (cellulose, hemicellulose, and lignin). In the case of the NCS bagasse, the lower lignin content indicated that the other fractions might be more available to be assimilated in pretreatment and digestion processes, among others. The content of extractives in both raw materials was considerable, especially in NCS bagasse. However, evaluating the feasibility of applying extraction processes for these components of interest is important for characterizing the extracts and reviewing the technologies that can be applied. In the PP case, different sections of the same residue types did not generate significant variations. Nevertheless, the pine species considerably affected some of the raw material components. For example, the extractives content is lower for different species than PP. On the other hand, although there is little literature on this specific residue, it can be compared to sugarcane bagasse (SCB). The variations in lignocellulosic (cellulose, hemicellulose, and lignin) were low, but the extractive and ash content changed. In general, the lignocellulosic raw materials are composed mostly of cellulose, hemicellulose, and lignin. Therefore, these components will be of great value when considering processes for using and transforming these raw materials.

**Table 5:** Comparison of obtained results with the composition of similar raw materials, %w/w

Raw material	Moisture	Ash	Extractives	Total fiber content			Reference
				Cellulose	Hemicellulose	Lignin	
PP	11.2 ± 0.22	0.41 ± 0.01	11.5 ± 0.24	33.0 ± 0.51	17.6 ± 1.05	26.3 ± 1.50	This work
PP	9.21	0.25	11.0	44.8	23.7	20.2	García et al. (2017)
PPB	4.00	3.29	17.7	23.0	27.0	25.0	Moncada et al. (2016b)
PPS	12.1	n.d	n.d	54.7	7.80	28.0	Sarria-Villa et al. (2018)
PM	n.d	13.1	2.63	75.2		25.1	Okon and Udoakpan (2019)
PR	n.d	n.d	3.20	39.7	18.1	26.6	Santos et al. (2022)
Bagasse from NCS production	7.26 ± 0.39	1.01 ± 0.03	20.4 ± 0.66	33.3 ± 0.28	19.9 ± 1.02	18.2 ± 0.35	This work
Bagasse from NCS production	n.d	5.50	9.50	38.0	34.0	13.0	Acosta et al. (2018)
SCB	n.d	4.00	5.90	40.6	20.9	17.4	Batalha et al. (2015)
SCB	n.d	0.80	n.d	51.8	27.6	10.7	Dorez et al. (2014)
SCB	n.d	8.80	2.70	38.5	27.8	17.7	Guilherme et al. (2015)
SCS	n.d	2.40	6.20	39.8	28.6	22.5	Oliveira et al. (2013)

*n.d.* not determined, *PP Pinus patula* wood chips, *PPB Pinus patula* bark, *PPS Pinus patula* sawdust, *PM Pinus massoniana* wood, *PR Pinus radiata* wood, *NCS* non-centrifuged sugar, *SCB* sugarcane bagasse, *SCS* sugarcane straw

### Total and volatile solids results

For PP, total solids (TS) and volatile solids (VS) were 88.99%w/w and 88.50%w/w, respectively, while for bagasse from NCS production, they were 90.28%w/w and 89.05%w/w. The TS and VS of the samples under analysis were used to calculate the BI. In this regard, the bagasse from NCS production and PP have BIs of 99.45% and 98.64%, respectively. Compared to other feedstocks frequently used in anaerobic digestion systems, their values are higher. For instance, plantain peel had a BI value of 78.20% and plantain pseudostem had a BI value of 87.44%, according to Piedrahita-Rodriguez (2020). Additionally, the reported BI for avocado peel was 97.77%. Relatively high BI values indirectly indicate the potential environmental problems generated by disposing of these raw materials in landfills due to the high levels of methane and leachate produced (Solarte Toro et al., 2023). Therefore, the values showed the potential of these raw materials to be used in anaerobic digestion processes to obtain biogas.

### Lignin characterization results

The results of the thioacidolysis experiment are illustrated in Table 6. In the case of PP, the analysis was performed on a pine species different from those reported in the literature. Thus, comparison was made with results obtained by other authors on other pine species (*Pinus radiata* and *Pinus pinaster*). Because pine is a softwood, lignin syringyl thioacidolysis monomers (S) were expected not to exceed 3% (Rolando et al. 1992). In contrast, a molar percentage of 17.5 ± 2.2 was obtained for S units in PP, suggesting that this sample contained some hardwoods. Indeed, the origin of the raw material could not be controlled since the company’s operators carried out the collection in Manizales and the company processes different woods. The hypothesis of a mixture of pine and hardwoods was further supported by the higher total monomers yield based on g Klason lignin (KL) calculated for the PP sample, compared to the value reported for pine species in the literature. Whereas the molar percentage of *p*-hydroxyphenyl monomers (H) was similar to other pine species (slightly higher), that of guaiacyl monomers (G) was lower than usually reported. In these comparisons, only those articles where thioacidolysis analysis was performed for samples without any pretreatment were considered. Nevertheless, variations in the results may be associated with storage conditions, mechanical grinding, and the origin of the raw material. These characteristics may somewhat affect the structure of the lignin, especially its content in β-*O*-4 linkages which are the prominent linkages cleaved by thioacidolysis. In the case of the bagasse from NCS production samples, a comparison of the results reported by other authors for sugarcane bagasse (residue for sugar value chain) indicated similar molar percentages of H, G, and S monomers, with a S/G ratio ranging from 0.87 to 1.82 (Table 6). The total monomer yield per g KL was higher than that reported by Yue et al. (2011) but similar to values obtained by the other authors.

**Table 6:** Determination of the main H, G, and S lignin monomers released by thioacidolysis of the lignocellulosic raw materials

Raw material	Total yield, µmol/g KL	H/G	S/G	Molar %H	Molar %G	Molar %S	Reference
PP	1304 ± 106	0.06 ± 0.003	0.23 ± 0.03	4.80 ± 0.30	77.7 ± 2.00	17.5 ± 2.20	This work
PR	814	0.03	0.00	3.15	96.8	0.00	Happs et al. (2021)
PR*	696	0.04	0.02	3.70	93.9	2.40	Harman-Ware et al. (2016)
	857	0.03	0.03	2.80	94.3	2.90	
PN	1017	0.03	0.00	2.46	97.5	0.00	Rolando et al. (1992)
Bagasse from NCS production	1193 ± 107	0.03 ± 0.002	1.82 ± 0.14	1.10 ± 0.01	35.2 ± 1.8	63.8 ± 1.8	This work
SCB	n.d	n.d	1.60	3.00	37.0	60.0	del Río et al. (2015)
SCB	1103	0.04	1.46	1.75	39.8	58.4	Miyamoto et al. (2018)
SCB	576	0.03	0.87	1.39	52.6	46.0	Yue et al. (2011)

*PP Pinus patula* wood chips, *PR Pinus radiata* wood, *PN Pinus pinaster* wood, *NCS* non-centrifuged sugar, *SCB* sugarcane bagasse, *KL* Klason lignin,* H* p-hydroxyphenyl monomer, *G* guaiacyl monomer, *S* syringyl monomer, *n.d*. not determined; *performed in two different laboratories, but same method

For the NCS bagasse samples, the results were compared with those reported by authors for sugarcane bagasse. These results show that the molar percentages of H, G, and S monomers are similar. Therefore, the S/G ratio was also similar, ranging from 0.870 to 1.82. The yields obtained with a g KL basis were higher than those reported by Yue et al. (2011) but similar to those of other authors (Table 6). Monomer S can be found for this type of lignocellulosic material, so the values found are expected.

Comparing the two raw materials, PP and bagasse, from NCS production, similar total thioacidolysis yields were obtained, but an eightfold higher S/G ratio typified the bagasse sample. The difference in the lignin composition of the two raw materials was also revealed by the determination of other compounds released by thioacidolysis (Table 7). As a structural characteristic of grass lignins, p-Coumaric and ferulic acid presence was observed only in the bagasse sample. The results of Table 7 indicate that an interesting value-added compound could be vanillin in the case of PP and p-Coumaric acid in the case of the bagasse from NCS production. These characterizations show the potential of lignocellulosic raw materials for extracting compounds of interest.

**Table 7:** Components present in lignin, identified through thioacidolysis

Component	PP, this work	Bagasse from NCS production, this work
5-OH guaiacyl units, µmol/g CW	0.90 ± 0.10	1.00 ± 0.10
Catechol units, µmol/g CW	1.10 ± 0.20	0.20 ± 0.03
Vanillin, µmol/g CW	3.60 ± 0.20	0.60 ± 0.10
*p*-Coumaric acid, mg/g CW	n.d	19.0 ± 2.7
Ferulic acid, mg/g CW	n.d	4.5 ± 0.5

*n.d.* not determined owing to very low relative amounts, *PP Pinus patula* wood chips, *NCS* non-centrifuged sugar, *CW* cell wall residues

### Geographical assessment

**Fig. 3:**
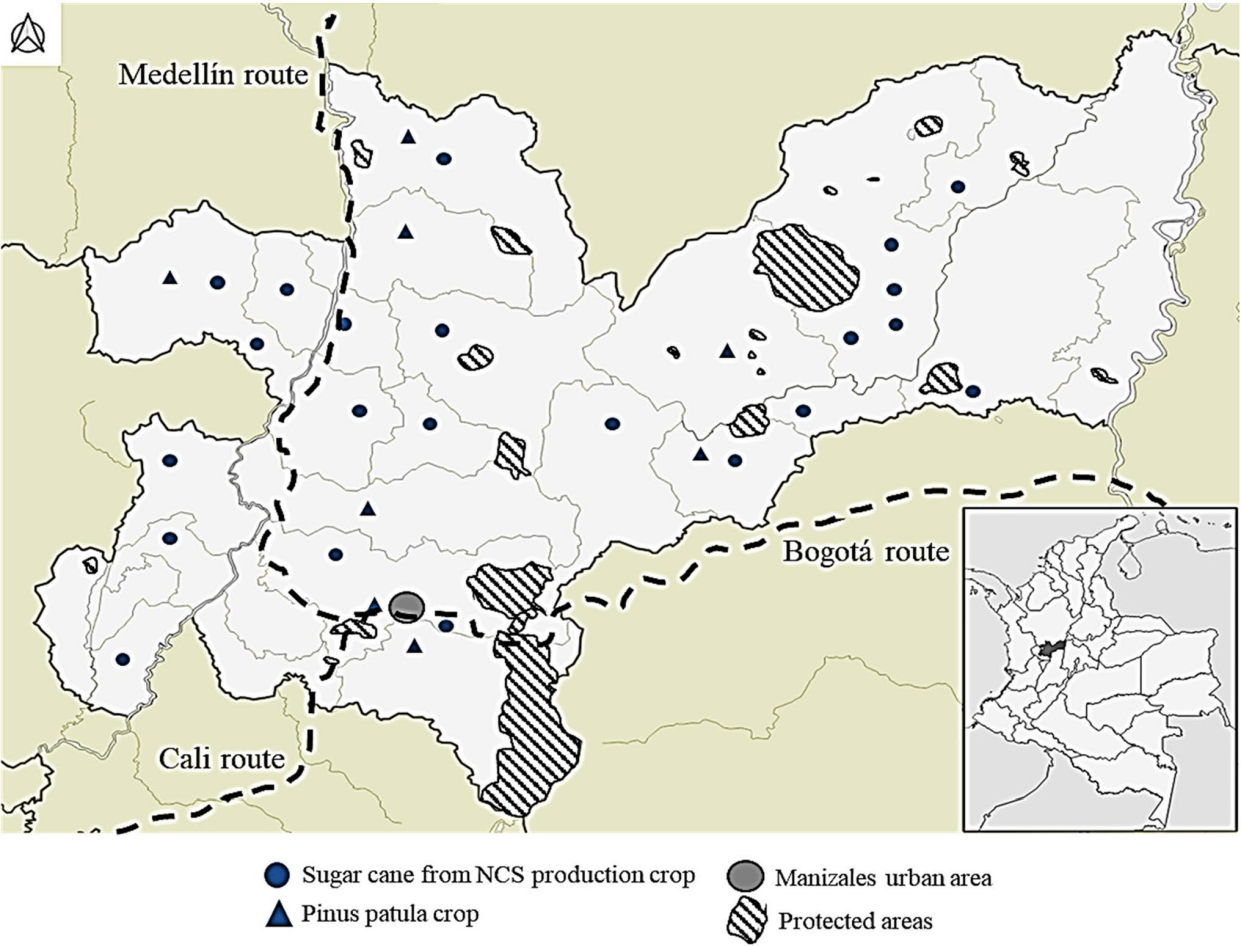
Caldas map including the sugarcane from non-centrifugal sugar (NCS) production crop, *Pinus patula* crop, and protected areas

A map was constructed for the geographic analysis (Fig. 3). It presented the approximate locations of the sugarcane from NCS production and *pinus patula* pine crops. These locations are similar for both crops, as the climatic and soil conditions benefit the production of these raw materials. The region of Samaná stood out, with a high presence of sugarcane from NCS production crops. As previously mentioned, the panela production process uses the bagasse to supply energy and complement it with wood residues. According to the locations of the sugarcane and pine crops, it is very likely that *pinus patula* is one of the woods used in this process. However, it should be noted that this use is partial, and there is still a problem with the final disposal of bagasse and pine. Caldas region has a road network connecting major cities such as Bogotá, Medellín, and Cali (Fig. 3). These road networks allow for a constant flow of inputs, raw materials, products, and goods between Manizales (capital of Caldas) and these major Colombian cities. Thus, this geographic analysis provides evidence of the possibilities of market routes for the products of the proposed transformation route schemes. It also complements the information for the geolocation analysis shown and discussed in Fig. 3.

The spatial distribution of precipitation is relevant for cultivation. In Caldas, the distribution of rainfall is very varied. In the region’s western part, it rains for 200 to 250 days a year, with temperatures between 20 and 24 °C. In the eastern zone, rainfall is approximately 100 days per year (Suares Hincapie et al. 2015). An area where rainfall is evenly distributed throughout the year benefits crop growth. Although sugarcane crops are tolerant to rainy periods and can withstand up to 14 days, increased moisture on the soil surface affects the appearance of pathogens. On the other hand, prolonged periods of more than 90 days without rain affect biomass production, which translates into a lower accumulation of sugars in the plant for harvesting. The combination of warm temperatures and a sufficient amount of rain contributes to the growth of sugarcane. Caldas has a tropical mountain climate with frequent rainfall throughout the year. Regular rainfall will help maintain adequate soil moisture and provide the water necessary for plant growth. Sugarcane requires a considerable amount of water for growth. In this area, where it rains between 200 and 250 days annually, water availability through this means can benefit the crops. However, considering the distribution and intensity of rainfall is important, as they can affect the development of sugarcane due to the amount of moisture in the soil. In some low and warm areas, the number of rainy days may be lower, but there is still significant rainfall during the year. In these areas, rainfall may be more concentrated in a specific season, such as the rainy season. In contrast, in mountainous and higher altitude areas, rainy days are likely to be more frequent due to the influence of humid winds from the Pacific Ocean. In these areas, rainfall is usually well-distributed throughout the year. The geographic analysis of this information contributes to the study of possible transformation routes because it conditions the presence of processing lines such as bagasse and wood drying. This stage is crucial to define the performance of the transformation process, which contributes to the economic (need for equipment) and technical (energy requirement for drying) pillars.

**Fig. 4:**
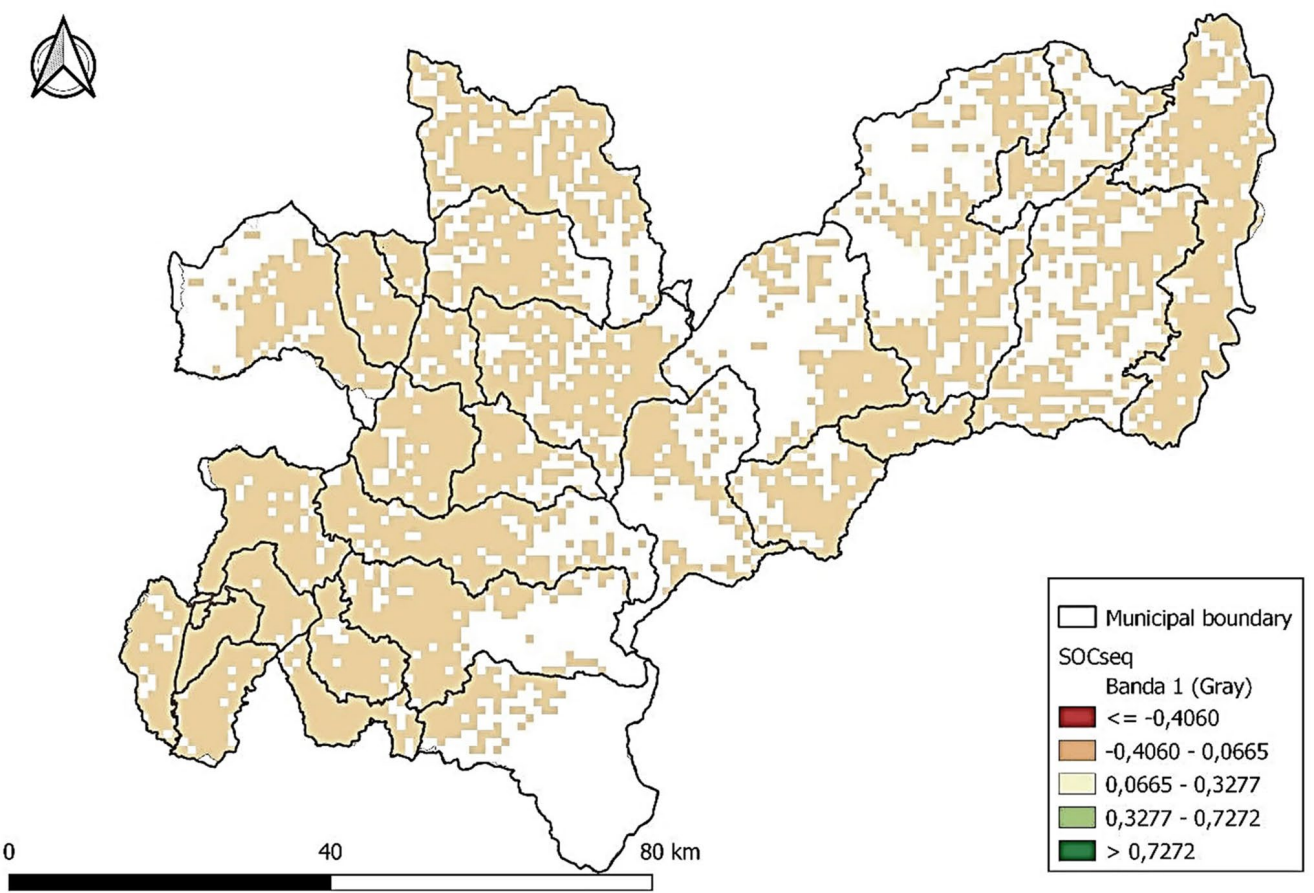
Soil organic carbon sequestration map, Caldas context. Units: kg C/ha

The interpretation of this information can be complemented with Fig. 4, which shows the impact of organic carbon sequestration and areas where agricultural practices and natural vegetation can interact favorably to increase organic carbon sequestration. The impact on the soil can also influence soil quality and carbon content. Some crops, such as cover crops, help increase soil fertility and water retention capacity. Still, other crops, such as intensive monocultures, can deplete soil nutrients and decrease the ability of the soil to store carbon (Lefévre et al. 2017). In contrast, warmer temperatures can accelerate the decomposition of organic matter, limiting the potential for carbon sequestration. However, organic matter accumulation in soils and ecosystems is more likely to occur in areas with cooler temperatures. Thus, the valorization processes of sugarcane crop residues should include obtaining products such as biofertilizers and biochar that can be applied to the soil and benefit the crop and its maintenance over time.

Regarding the residues of these crops, this geographic analysis allowed to identify several aspects. First, in terms of the NCS bagasse, taking advantage of the days without rain to dry this residue is possible, which would benefit the energy efficiency of the sugarcane mills for its adequate use in the burning process. Thus, the use of wood from various species is reduced. The transformation routes of both residues allowed the use of these raw materials and their return to the value chain. The processes to be obtained by applying the methodologies will reflect the integral use of raw materials. However, it is necessary to consider geographic locations as potential points to locate the processes. In the case of Caldas, the areas available for cultivation are scarce (as evidenced from the maps) and implementing new processing could increase the availability of raw materials. Therefore, evaluating the conditions of these points and the production capacities was necessary. For example, locations with a high rainfall level should be considered, as this reduces water consumption in the cultivation stage. In the life cycle of raw materials, it is also necessary to identify areas whose agronomic conditions are optimal and guarantee continuity in cultivation, for example, those with a high level of carbon sequestration (mitigating environmental impacts) or where protected areas are not compromised. Thus, geographic analysis plays a crucial role in the life cycle analysis of raw materials, providing limitations or restrictions for valorizing any of the products in the value chain. The maps constructed showed the importance of knowing the context and geographic level of the study area for decision-making regarding implementing new technologies.

### Strategy 1: criteria model proposal

**Fig. 5:**
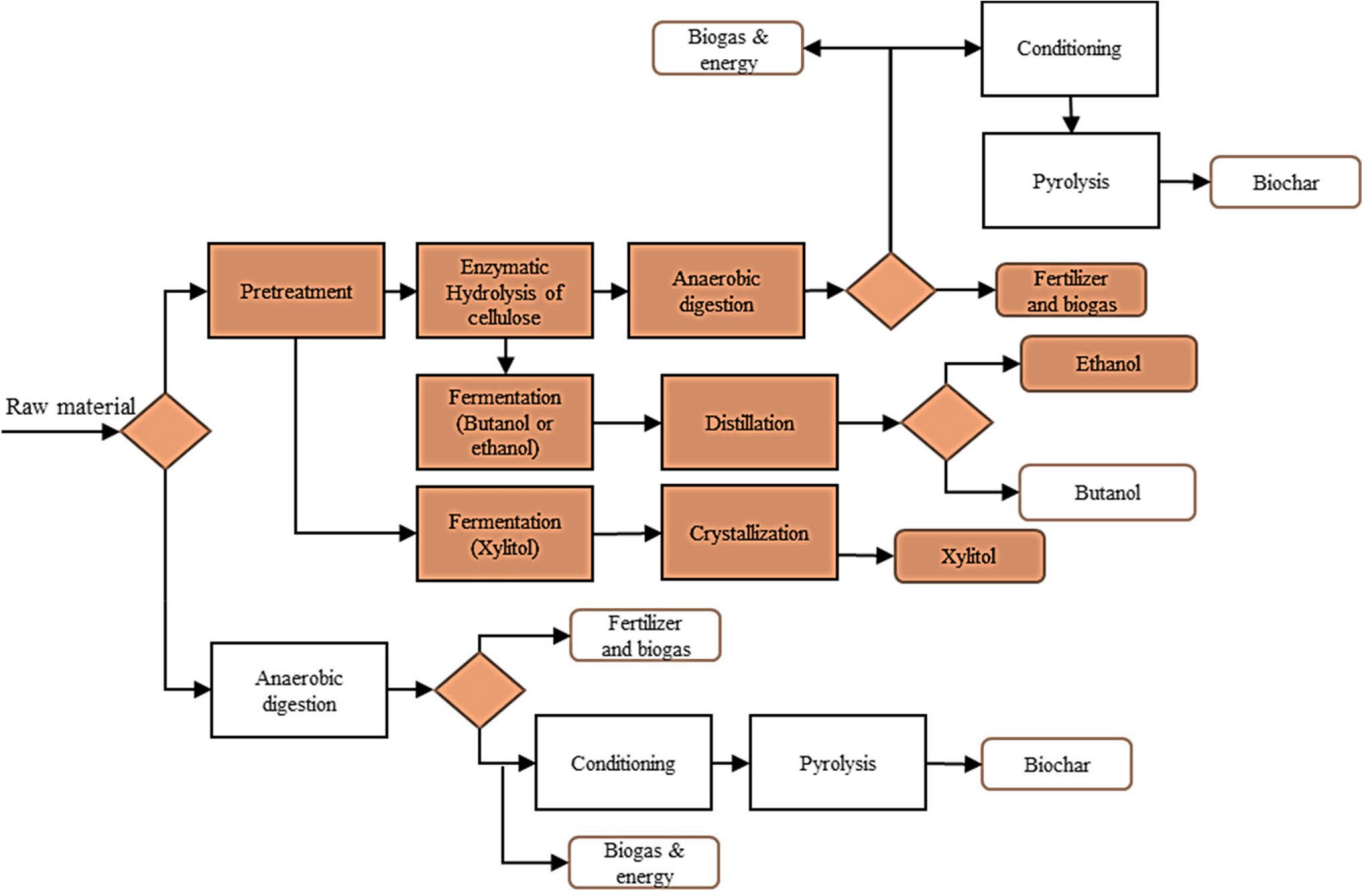
Superstructure of different biorefinery schemes applying the graphical composition model, for the use of *pinus patula* wood chips (PP) and the bagasse from non-centrifugal sugar (NCS) production

Applying the transformation route selection strategy showed that the most important fractions considered in the scenarios corresponded to cellulose, hemicellulose, and lignin. These three fractions coexist in different ratios depending on the feedstock, and the presence of one or the other may lead to limitations in the sustainability assessment of the transformation processes. For example, the lignin content present in the feedstock relative to the content of the other fractions can limit accessibility, leading to restrictions on process yields such as pretreatments, fermentations, or digestions. Therefore, if the composition of these fractions is normalized for both raw materials, the ratio of cellulose and hemicellulose to lignin content was 2.93 for NCSB and 1.92 for PP. In both cases, the defined level was 2, corresponding to well-established fermentation processes (TRL > 7) and anaerobic digestion. Fermentative processes within this classification allow obtaining ethanol, xylitol, and butanol mainly, so these two schemes are considered. Based on the composition of the lignin monomers identified for the raw materials, transformation processes were not selected since the content of these molecules needed to be higher for the technical requirements. Considering the composition of extractives, a high content is identified for the bagasse from NCS production, which could indicate the feasibility of its utilization. However, specific identification of the major metabolite in the extractives and its proportion (dilution issue) is required to define its interest. Thus, since this information is not available, the processing of this fraction could not be assured. Regarding the biodegradability of the raw materials, the BI value was greater than 70% so that anaerobic digestion processes could serve this purpose. The products of anaerobic digestion can be used in different ways. Therefore, two possible routes were established: (i) generating biogas and fertilizer from the digestate or (ii) obtaining biogas and energy by co-generation and pyrolysis of the digestate to obtain biochar. Finally, the fats and protein content in the analyzed raw materials was insufficient to evaluate their valorization. Considering this analysis, the biorefinery scheme superstructure of Fig. 5 was proposed. The underlined scheme is the proposed biorefinery case for both raw materials, which was evaluated in terms of sustainability.

### Strategy 2 for the selection of the best routes

#### Step 1

The experimental section’s compositional results defined that the main components are the lignocellulosic content (cellulose, hemicellulose, and lignin fractions). The sustainability goal was defined, which was established according to the strategy for the case of both raw materials considering those mentioned above. The sustainability objective is “To find schemes of transformation routes for the bagasse from NCS production and PP generated in the industries of Caldas to reduce the number of residues disposed in the region.”

#### Step 2

Regarding the composition of the raw materials, only those technologies where the cellulose, hemicellulose, and lignin fractions are fully exploited were selected. Furthermore, since high TRLs are required in the region (greater than 7), the processes were filtered according to Fig. 4, available in the Ortiz-Sánchez and Cardona Alzate (2022) strategy. Table 8 presents the technologies selected with this first filter for each fraction. For cellulose, the most relevant technologies for upgrading are ethanol, butanol, lactic acid, and levulinic acid, while for hemicellulose, xylitol and pentane were chosen. Furthermore, for these processes, pretreatment steps were also selected for the production of the precursor platforms. These pretreatments selected dilute acid, hot liquid water, and alkaline. Finally, the valorization of the lignin fraction originated the selection of phenolic compounds, synthesis gas, biogas, energy, and biochar technologies.

**Table 8:** First technologies selection based on the composition of the raw materials

Fraction	Upgrading technologies	Pretreatment
Cellulose	Ethanol, butanol, lactic acid, levulinic acid	Dilute acid, liquid hot water, alkaline
Hemicellulose	Xylitol, pentane	
Lignin and others	Phenolic compounds, syngas, biogas, energy, biochar	N.A

*N.A.* not applicable

#### Step 3

The previous bioprocesses were filtered again by evaluating technical (mass and energy), economic, and environmental indicators. According to the sustainability objective defined, the bioprocesses selected were those in which CapEx and OpEx were low and had a low environmental impact. In this, the routes and indicators available in Table 1 of the Ortiz-Sánchez and Cardona Alzate (2022) strategy were studied. Lactic and levulinic acids were discarded for the cellulose platform due to its economic and environmental indicators (high compared to the other processes). In the case of hemicellulose, the pentane production process was dispensed because it has a higher environmental indicator than xylitol. Likewise, compared to diluted acid, alkaline pretreatments, and liquid hot water represent the highest economic indicators and are therefore discarded. In addition, at the technical level, the pretreatment selected was a standardized process with established optimizations. Finally, in the case of lignin, although it is possible to extract phenolic compounds of commercial value for both raw materials, the technology does not represent the best economic or technical indicators. Therefore, this bioprocess was discarded, and the remaining processes (syngas, biogas, biochar, and energy) were kept in this selection.

#### Step 4

**Fig. 6:**
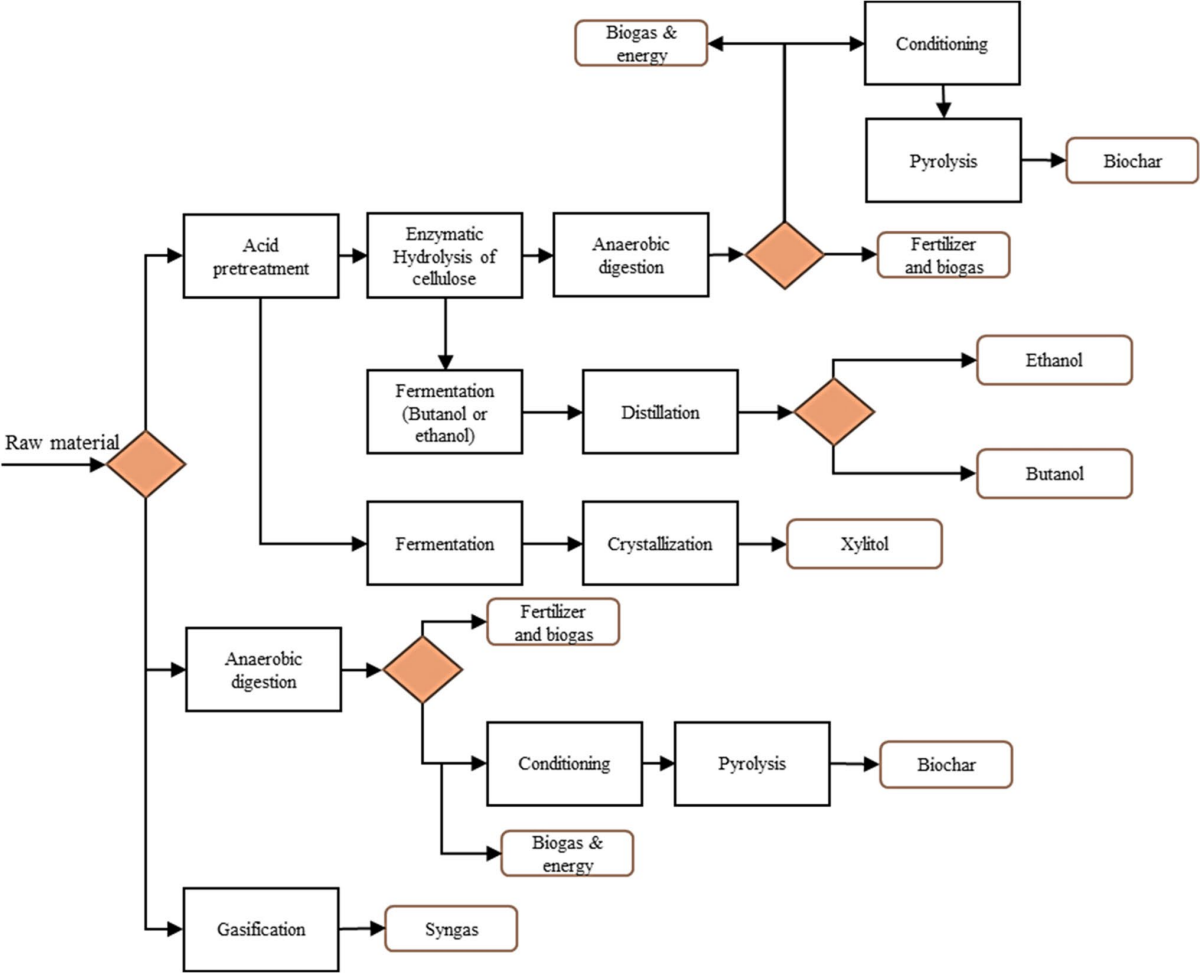
Superstructure of different biorefinery schemes applying the strategy of Ortiz-Sánchez and Cardona Alzate (2022), for the use of *pinus patula* wood chips (PP) and the bagasse from non-centrifuged sugar (NCS) production

Finally, based on this strategy, a superstructure was defined as depicted in Fig. 6. This superstructure shows all the possible options to be considered in biorefinery schemes. The proposed schemes allowed an integral use of all the fractions of raw materials. It could indicate that the environmental impact was reduced by generating less direct waste for disposal in landfills or burning. However, these schemes should be analyzed in the future (accompanied by process design strategies and sustainability evaluation) to ensure viable selection. Comparing the proposed superstructures, the transformation route selection strategy proposed syngas as an alternative for PP and the bagasse from NCS production use. However, applying the criteria model, this product was discarded. Nevertheless, the other schemes remained similar for both studies and feedstocks. Therefore, the transformation routes defined by both methodologies allowed the proposal of biorefinery schemes for using these lignocellulosic biomasses.

### Pretreatment selection

The composition of the hydrolysate based on xylose, glucose, furfural, and HMF can be seen in Table 9. Similar results can be found in the case of dilute sulfuric acid for PP, the bagasse from NCS production, and NaOH pretreatment. The lowest values found were for TFA pretreatment. Thus, the best pretreatment condition was the diluted sulfuric acid. This pretreatment was selected as the best case for transforming the PP and the bagasse from NCS production according to the defined criteria model.

**Table 9:** Pretreatment results for each case

Raw material: *Pinus patula* wood chips (PP)				
Pretreatment	Xylose (g/L)	Glucose (g/L)	Furfural (g/L)	HMF (g/L)
Dilute sulfuric acid	21.2	28.54	0.02	0.02
Dilute TFA	0.020	0.019	n.d	n.d
Dilute NaOH	18.6	19.5	0.03	0.02
Raw material: bagasse from non-centrifuged sugar (NCS) production				
Pretreatment	Xylose (g/L)	Glucose (g/L)	Furfural (g/L)	HMF (g/L)
Dilute sulfuric acid	15.1	31.5	0.01	0.02
Dilute TFA	0.055	0.021	n.d	n.d
Dilute NaOH	12.1	22.2	0.03	0.03

*n.d.* not determined, *TFA* trifluoracetic acid, *HMF* hydroxyethyl furfural

### Biorefinery evaluation

#### Techno-economic assessment

**Fig. 7:**
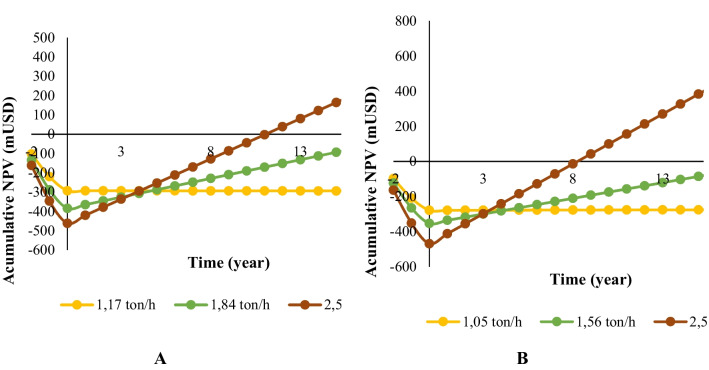
Net present value for *pinus patula* wood chips (PP) (**A**) and the bagasse from non-centrifuged sugar (NCS) production (**B**) biorefinery schemes

The technical metrics evaluated in this section were PMI and RMI. For the case of PP, the biorefinery scheme obtained a PMI and RMI of 48.2 and 1.16, respectively, and for the bagasse from NSC production the values were 44.1 and 1.16. Both cases have advantages regarding renewable materials susceptible to transformation since all the important fractions of the biomass are used. The OpEx in the PP and the bagasse from NCS production cases was USD 3.51 and USD 13.4, respectively. Chemical reagents significantly influenced on the OpEx in the two schemes (32.1 and 63.7% for the PP and the bagasse from NCS production cases). Enzyme consumption in the enzymatic hydrolysis of the bagasse from NCS production and PP was the most representative. Using sulfuric acid, lime, and process water consumption did not increase the costs of chemical reagents compared to the schemes. Maintenance costs, capital depreciation, fixed charges, general and administrative costs, and insurance and taxes in PP cases had a lower contribution than the bagasse from NCS production. Indeed, maintenance and plant overhead costs contributed 9.47% and 8.26% in the bagasse from NCS production biorefinery because the fixed capital investment was higher than that in the PP case. The CapEx for the PP and the bagasse from NCS production biorefinery schemes was mUSD 16.8 and mUSD 19.0, respectively. The equipment cost in both schemes represented 23% of CapEx. The bioethanol and xylitol production stages represented 17% and 7% of the equipment cost for the PP case, respectively. For the bagasse from the NCS production scheme, the bioethanol and xylitol production stages contributed significantly to the equipment cost (53% and 23%, respectively). Total direct plant cost (i.e., equipment installation, instrumentation, control, piping installation, electrical installation, buildings including services, yard improvements, and service facilities installation) represents 56% of CapEx for all cases. Figure 7 depicts the NPV in the economic evaluation. The scale in which the equilibrium point was presented was 1.17 ton/h and 1.05 ton/h for PP and bagasse from NCS production, respectively. The MPSEF was 1.84 ton/h and 1.56 ton/h for the PP and the bagasse from NCS production schemes. In this sense, the biorefinery schemes could be applied in the study region of this work because of the processing scale available in the zone. The indicators PBP and TR values were 13 and 5 for the PP case and 9 and 5 for the NCS bagasse case.

#### Environmental assessment

The results of system boundaries and LCA inventories for PP and NCS production schemes are shown in Supplementary Material 3 (SM3). Considering the information given in SM3, the results of the environmental metrics were 5.85 kgCO_2_eq and 1.29 kgCO_2_eq for CF for PP and the bagasse from NCS production schemes, respectively, and 17.7 m^3^/kg and 0.83 m^3^/kg for WD. For the two calculated metrics, it can be seen that the environmental impact for the case of PP was higher than that for the case of the bagasse from NCS production. The category with a significantly higher impact was water depletion for the PP scheme and carbon footprint for the bagasse from the NCS production scheme. The energy and service fluid consumption make these categories highly impacted. Some studies regarding the valorization of lignocellulosic raw materials are available. For example, Botero Agudelo et al. (2011) calculated the environmental impact of ethanol production from cassava, in 19.30 kg CO_2_ eq/kg ethanol. This high value refers to the low ethanol conversion obtained, thus generating large amounts of effluents and emissions.

##### Geolocation part

The locations with the lowest average travel times are located in the western and southern part of Manizales centre (around 183 min). Distances were found to be around 202–384 km. The best locations according to this information are in the west area of Manizales centre. Table 10 shows the carbon footprint for each scenario considered (local, regional, and national). It is evident that the higher the distance, the higher the carbon footprint in the Cali and Medellin distribution zones. Furthermore, locations with the lowest CO_2_ emissions are near the feedstock centre because near to 60% of the carbon footprint is produced during the transport from the feedstock centre to the potential biorefinery locations. Thus, the best location is 2.51 km at the east of Manizales municipality. It is the nearest point to the feedstock centre. This point is the best option in all the schemes studied.

**Table 10:** Carbon footprint for the best location of the biorefinery schemes

Scenario	Carbon footprint
Local distribution (Manizales)	0.75-ton CO_2_-eq/year
Regional distribution (Cali and Manizales)	2.05-ton CO_2_-eq/year
National distribution (Cali, Medellín, and Manizales)	6.85-ton CO_2_-eq/year

#### Social assessment

The minimum wage is 287 USD/month (1 USD = 4029 COP, 15/08/2023). The living wage of a single person in Colombia in 2023 was estimated at 546 USD/month. Thus, the M/L ratio was 0.72 for PP and 0.69 for the bagasse from NCS production. The indicator value is low since more than 59% of the Colombian working population earns a minimum wage or even less. Thus, a high share of the Colombian people needs more income to have a decent quality of life. Consequently, the sensitivity analysis was conducted to determine the resilience of the proposed biorefineries, considering an increase in the minimum wage. In terms of the local employment indicator, it was determined that the total days worked per employee considered 17 holidays, 15 vacation days, 2 days off per year for family time, and 1.12 days off per week (with total hours of 47 h per work week). Therefore, the total work per employee calculated was 274 days per year. The ratio between the total work time per year of the plant and the work time per year of an employee determined the number of employees per area. This value resulted in five employees per process area plus one additional employee for an additional labor force, i.e., six operators per process area. Besides, three supervisors are required per shift. Therefore, a total of 9 employees are required per process area. The total number of employees calculated for the PP biorefinery was 21, and 24 for the NCS bagasse biorefinery.

#### Sustainability index

The sustainability index was estimated based on the results presented in Table 11. The metrics were calculated according to the economic feasibility scale (2.50 ton/h). The bagasse from NCS production scheme that produced xylitol, bioethanol, and biogas presented the best sustainability index (60.91%). The sustainability index result allows proposing the biorefinery as a good alternative for more study and future implementation. The economic and environmental dimensions were the most influential factors in the global sustainability score, contributing 19.7% and 24.5% to the total sustainability score. In contrast, the technical and social dimensions had the lowest contribution, with 7.18% and 9.50%, respectively. Then, more efforts should be made to increase this dimension’s technical and social score. Biorefinery configurations producing xylitol and bioethanol have shown good economic and environmental performance.

**Table 11:** Results of the sustainability index assessment

Dimension	Indicator	PP biorefinery	Bagasse from NCS production biorefinery
No normalized			
Technical	PMI	48.2	44.1
	RMI	1.00	1.00
Economic	PBP	13.0	9.00
	TR	5.00	5.00
Environmental	CF	5.85	1.29
	WD	17.7	0.83
Social	(M/L) max	0.71	0.69
Normalized values			
Technical	PMI	0.52	0.56
	RMI	0.01	0.01
Economic	PBP	0.37	0.58
	TR	1.00	2.31
Environmental	CF	0.73	0.96
	WD	0.12	1.01
Social	(M/L) max	0.42	0.38
Sustainability index	SI	44.8	60.9

*PP Pinus patula* wood chips, *NCS* non-centrifuged sugar, *PMI* process mass intensity, *RMI* renewable material index, *PBP* payback period, *TR* turnover ratio, *CF* carbon footprint, *WD* water depletion, *M/L* minimum-to-living wage ratio, *SI* sustainability index

The results obtained by applying the transformation route selection strategy have some limitations. Mainly, the accuracy of the route obtained depends on the quality of the information provided to feed the strategy. For example, if the raw material characterization data are taken from experiments, the accuracy of the route selection is likely to be much closer to a feasible process. However, the strategy has the advantage that it can be applied with literature data, for those cases where the potential of a feedstock needs to be analyzed and initial processing decisions need to be made.

The raw material selection strategy is a very useful tool. Additionally, considering sustainability aspects makes it more appealing and necessary according to current developments in the industry. However, it is encouraged that these investigations continue to be complemented with new tools and approaches, as is the case of the work done by Aghbashlo et al. (2022). It distinguishes sustainability assessment from an exergetic point of view. If this study were to be applied to the sustainability assessment of the transformation route selected through the strategy, it is expected that the results would not vary much from those already considered in this paper but would be an interesting complement and provide a different point of view for the debate in relation to this analysis.

## Conclusion and prospects

The proposal of biorefinery schemes was possible by implementing a systematic criteria model strategy derived from a rigorous analysis of the chemical composition of lignocellulosic biomasses. A comprehensive evaluation of feedstock composition allowed for finding similarities and differences crucial in guiding the delineation of potential transformation routes. Biorefineries are the most comprehensive schemes to cover each platform susceptible to transformation. Geographical analysis served as a contextual assessment of raw material life cycles, revealing insights into crop location rationale, as depicted in constructed maps, along with identified bottlenecks and improvement opportunities. Applying a bioprocess selection strategy compared to the criteria model affirmed that feedstock composition is the foremost criterion for biomass valorization in biorefineries.

Based on the criteria model strategy, the sustainability analysis demonstrated the feasibility of proposing viable schemes implementable within a study region. Despite similar chemical compositions in assessed feedstocks, distinct economic and environmental indicators among biorefinery schemes underscored variations in the sustainability index. Currently, obstacles to the transition toward sustainable production in biorefineries lie in technological maturity (technological readiness level (TRL)) and the economic viability of processes. Future research could include an exploration of market dynamics and regional policies within the model, offering insights into significant limitations and restrictions influencing the selection of transformation routes. For example, the use of renewable materials must be permitted and monitored by government agencies. That is why, perhaps, decision-making regarding their valorization may be limited.

## Supplementary Information

Below is the link to the electronic supplementary material.

Supplementary file1 (DOCX 29 KB)

Supplementary file2 (DOCX 48 KB)

Supplementary file3 (DOCX 296 KB)

## Abbreviations

NCS: Non-centrifuged sugar
PP: *Pinus patula* Wood chip
GHG: Greenhouse gas
SOCseq: Soil organic carbon sequestration
OSM: OpenStreetMap
CH/L: Ratio of cellulose and hemicellulose with lignin
TS: Total solids
VS: Volatile solids
BI: Biodegradability index
TRL: Technological readiness level
DNP: National Planning Department
TFA: Trifluoroacetic acid
HPLC: High-performance liquid chromatography
NRTL-HOC: Non-random two liquids and Hayden-O’Connell
H: P-Hydroxyphenyl monomers
G: Guaiacyl monomers
S: Syringyl monomers
KL: Klason lignin
PMI: Process mass intensity
RMI: Renewable material index
NPV: Net present value
PBP: Playback period
TR: Turnover ratio
CapEx: Capital expenditures
OpEx: Operational expenditures
MPSEF: Minimum Processing Scale for Economic Feasibility
EPA: US Environmental Protection Agency
LCA: Life cycle assessment
CF: Carbon footprint
WD: Water depletion
M/L: Minimum-to-living wage ratio
SI: Sustainability index
TcIn: Technical indicators
EcIn: Economic indicators
EnIc: Environmental indicators
SIn: Social indicators

## Units

ton: Tones
Mton: Million tones
h: Hour
yr: Year
m: Meters
km: Kilometers
mg: Miligrams
g: Grams
v/v: Volume/volume percentage
M: Molar
L: Liter
