# Supplementary material for: Strategy for the analysis of lignocellulosic biomass to select a viable transformation route in the Colombian context
Source: Environ Sci Pollut Res Int. 2024 May 2;32(48):27741–62. doi: 10.1007/s11356-024-32975-x (PMC12696050; doi:10.1007/s11356-024-32975-x)
Supplement: Supplementary file 1 — Supplementary file1 (DOCX 29 KB) [file 11356_2024_32975_MOESM1_ESM.docx]

**Supplementary Material 1**

**Title:** Analysis of lignocellulosic biomass composition for selection of transformation routes in the Colombian context

**Authors:** Sara Piedrahita-Rodríguez^1^, Andrés-Felipe Alzate-Ramírez^1^, Stéphanie Baumberger^2^, Laurent Cézard^2^, Mariana

Ortiz-Sánchez^1^, Diego Alexander Escobar García^3^, Ana María Zetty Arenas^1^, Konstantinos Moustakas^4^, Carlos Ariel Cardona Alzate^1*^

^1^Instituto de Biotecnología y Agroindustria, Departamento de Ingeniería Química, Universidad Nacional de Colombia, Manizales, Caldas, Zip Code: 170003, Colombia

^2^Institut Jean-Pierre Bourgin (IJPB), INRAE, AgroParisTech, Université Paris-Saclay,78000 Versailles, France

^3^Universidad Nacional de Colombia, Sede Manizales, Facultad de Ingeniería y Arquitectura, Departamento de Ingeniería Civil, Grupo de Investigación en Movilidad Sostenible (GIMS), Campus La Nubia, Manizales, Caldas 170003, Colombia

^4^National Technical University of Athens, Unit of Environmental Science & Technology. School of Chemical Engineering, Greece

***Corresponding author:** ccardonaal@unal.edu.co

**SM1. Characterization methods**

Initially, the raw materials were dried and ground. Next, they were sieved to 40 mesh to correctly characterize them according to international standards. All the determinations were performed in triplicate.

1. **Moisture**

The MOC-120H balance was used for moisture determination. Approximately 0.5 g of the raw material was placed on the balance and heated at 105°C to constant weight.

1. **Extractives**

10 g of raw material was taken and placed in extraction bags previously weighed. They were taken to the Soxhlet extraction system. They were extracted with water (270 ml in a flat-bottomed balloon), leaving the system for approximately 24 hours. Finally, the extraction bag is dried and weighed to be taken to the same system but with ethanol extraction. The dry samples free of extractives were stored for later analysis [1-2].

1. **Ash**

A muffle was used to determine the ashes of the raw materials. Calcined porcelain crucibles were used, and 0.5 g of biomass was subjected to the heating ramp up to 575°C for 3 hours. The crucibles with the ashes were cooled and weighed [3].

1. **Holocellulose**

First, a thermostatted bath was preheated to 70°C. 2.5 g of extractive-free raw material was placed in a 250 mL Erlenmeyer, and 80 mL of hot water, 0.5 mL of acetic acid, and 1 g of sodium chlorite were added. Next, a 50 mL Erlenmeyer flask was placed over the sample to retain the reaction's gases. This setup was placed in the thermostatted bath at 70°C. After 1 hour, 1 mL of acetic acid and 1 g of sodium chlorite were added with gentle stirring. Acetic acid and sodium chlorite were added six more times (every hour). After the last addition, the assembly was left for 24 h at heating at 70°C. Finally, the samples were filtered under a vacuum (first with acetone and then with plenty of distilled water). The solids were dried and weighed. This way, the holocellulose content is determined [4].

1. **Cellulose**

2 g of the above holocellulose sample was taken to a 250 mL Erlenmeyer, and 10 mL of 17.5% NaOH was added. The sample is brought to a thermostatted bath at 20°C. At 5-min intervals, five more mL of 17.5% NaOH was added for 45 min. Subsequently, 33 mL of water was added, leaving the system for an additional 60 min. Finally, the cellulose was vacuum filtered by initially washing with 100 mL of an 8.3% NaOH solution, then with distilled water, then with 15 mL of 10% glacial acetic acid, and finally with plenty of distilled water. The sample was dried and weighed to determine the cellulose content [4].

1. **Hemicellulose**

To determine hemicellulose, it is sufficient to know the holocellulose and cellulose content and apply equation **1**.

| $Holocellulose=Hemicellulose+Cellulose$ | **Eq. 1** |
| --- | --- |

1. **Lignin**

Approximately 150 mg of the sample was weighed and transferred to 100 mL beakers. Then, 2.5 mL of 72% w/w H_2_SO_4_ was added. The sample with the acid was left to react for 2 hours at room temperature, occasionally homogenizing with a small glass stirrer (every 20 min). In the end, the sample was transferred to 250 mL round-bottomed flasks and 38 mL of deionized H_2_O was added, diluting to a final concentration of 5% w/w H_2_SO_4_. This addition of water must be done carefully since all the volume must be used to help transfer all the solid samples. Subsequently, the beakers with the sample and the diluted acid were placed in a sand bath, previously heated, and with the cooling system running. This assembly was left to react for 3 hours. It was monitored that the sample did not remain adhered to the walls of the ball without having contact with the acid. If this occurs, the balloon is gently shaken (with thick gloves and caution) to homogenize the sample [5].

Once the time has elapsed, the balloons can cool to room temperature. Previously, the crucibles with glass filters were weighed, and each sample was filtered with the help of a vacuum system that sped up the process. During filtration, deionized water can be added to wash the balloon and ensure that all the solid fraction remains inside the crucible. Next, the crucibles are placed in an oven at 105°C overnight. Finally, the dried crucibles are allowed to cool at room temperature and in a desiccator and then weighed. This last value will be used for ash determination. The lignin Klason and ash content is calculated using equations **2-**

| $Klason content with ash \left( \% \right)=\frac{Residue \left( mg \right)}{Initial sample \left( mg \right)}*100$ | **Eq. 2** |
| --- | --- |
| $Ash content \left( mg \right)=Dry crucible after incineration \left( mg \right)-crucible with filter \left( mg \right)$ | **Eq. 3** |
| $Klason content without ash \left( \% \right)=\frac{Residue without ashes \left( mg \right)}{Initial sample \left( mg \right)}*100$ | **Eq. 4** |
| $Residue \left( mg \right)=crusible with filter and insoluble residue \left( mg \right)-crucible with filter \left( mg \right)$ | **Eq. 5** |
| $Residue without ashes \left( mg \right)=Residue \left( mg \right)-Ash content \left( mg \right)$ | **Eq. 6** |

1. **Thioacidolysis**

Approximately 10 mg of the sample was transferred to capped test tubes. Hereafter, all reagent additions were performed in the extraction cabinet. First, 100 µg of internal standards (C19, C21, and C22) of known concentration (2 mg/mL) were added to each tube. Next, the thioacidolysis reagent (10 mL ethanethiol and 2.5 mL BF_3_EtO) was prepared in a 100 mL volumetric flask, gauged with dioxane. Immediately after the reagent was prepared, 7 mL was added to the samples and capped tightly because dioxane ad ethanethiol are volatile substances. Previously, an oil bath was heated and maintained at 100°C, and all tubes were brought into it and left for 4 hours. After this time, the tubes are removed and allowed to cool to room temperature. The liquid level of the samples should not have changed considerably, nor should the solutions have been colored black. Otherwise, this would indicate side reactions and contaminations, and the determination cannot be performed correctly. Subsequently, 7 mL of a 0.2 M NaHCO_3_ solution is added to each tube and the reaction mixture is acidified with 0.7 mL of a 6 M HCl solution. For phenolic compounds extraction, 7 mL of dichloromethane was added to each tube, originating two phases. The organic phase is dried with Na_2_SO_4_ and transferred to a ground ball for rotary evaporated to approximately 1 mL [6].

- 1. **Chromatography-mass spectrometry analysis**

An aliquot (5 µL) of each sample was added to a new vial (with inserts) before addition of 100 µL of the silylating reagent BSTFA (N,O-bis(trimethylsilyl)trifluoroacetamide) and 10 µL of pyridine. The samples were left to react for 2 hours (ideal time for the silylation process). Subsequently, they were injected into the GC-MS Varian 4000 instrument (Varian, Les Ulis, France) with an autosampler. The splitless injector (270°C) and an ion trap mass spectrometer are coupled to the system. The carrier gas used was helium (N60). A VF-1 ms 30 m x 0.25 mm polydimethylsiloxane capillary column, with a temperature program (45°C to 180°C at 30 °C/min and 180°C to 245°C at 2 °C/min), was used. The column flow rate was 1 mL/min. The lignin monomers H, G, and S concentrations were calculated in µmol/g, based on the chromatograms reconstructed on the ions with m/z 239, 269, and 299, respectively.

The calculations for the yields of each monomer and other components identified in the chromatograms are described below. Initially, the starting point is the calculation of the column coefficient, which is given by equation **7**. The internal standard to be used must first be corroborated. For this reason, a mixture of known concentrations of C_19_, C_21_, and C_22_ was prepared in the sample treatment. To verify that the standards and the column work correctly, it is necessary to perform the area reading in the chromatograph for each of these standards, locating the 57+71+85 ions. The ratio between the areas of C_19_ and C_21_ must be close to 1. Otherwise, the column may be contaminated, or the standards may not be prepared at the necessary concentration conditions. After this corroboration, the calculation of $coeff$ can be performed without problem, considering only one internal standard (it can be C_21_).

| $coeff=\frac{mg_{IS}*k*1\cdot{10}^{6}}{mg_{inicial}*{Surf}_{IS}}$ | **Eq. 7** |
| --- | --- |

Where, $mg$: mass, in milligrams; $IS$: Internal standard (mass=0.2 mg); $Surf_{IS}$: Area of internal standard; $k$: column response coefficient, fixed at 0.85.

The lignin monomers commonly identified in thioacidolysis are the *p*-hydroxyphenyl (H), guaiacyl (G), and syringyl (S) monomers. As mentioned in the introduction, each structurally represents lignin from lignocellulosic biomasses. Equations **8** and **9** show the calculation for monomer yields and total yields, respectively (per gram of cell wall (CW), or biomass). These yields can also be expressed with respect to the Klason lignin (KL) content in the sample (see equation **10**).

| $Monomer yield \left( \mu mol/g CW \right)=\frac{Surf_{monomer}*coeff}{molar mass_{monomer}}$ | **Eq. 8** |
| --- | --- |
| $Total yield \left( \mu mol/g CW \right)=H_{yield}+G_{yield}+S_{yield}$ | **Eq. 9** |
| $Total yield \left( \mu mol/g KL \right)=\frac{Total yield \left( \mu mol/g CW \right)}{\%KL}*100$ | **Eq. 10** |

It is also possible to find reported monomers as molar percentages (CW or KL basis). For this, equation **11** can be applied.

| $\%Monomer=\frac{Monomer_{yield} \left( \mu mol/g CW \right)}{Total yield \left( \mu mol/g CW \right)}$ | **Eq. 11** |
| --- | --- |

Finally, the ratio between H/G and S/G were calculated, dividing the ratios of each monomer. It is a simple way to compare and express the results according to thioacidolysis protocol. In this work, the yields of the monomers H, G, and S were determined, in addition to the monomers shown in **Table 1**, where the values of the ions and molar masses taken for the calculations can be seen.

**Table 1. Ions and molar masses for monomers identified in samples.**

| **Monomer** | **Ion chromatograms reconstructed at m/z** | **Molar mass** |
| --- | --- | --- |
| **H** | 239 | 316 |
| **G** | 269 | 346 |
| **S** | 299 | 376 |
| **5-OH guaiacyl units** | 357 | 362 |
| **Catechol units** | 327 | 332 |
| **Vanillin** | 269 | 258 |
| **Syringaldehyde** | 299 | 288 |
| ***p*-Coumaric acid*** | 308+293 and 309+265 | 164 |
| **Ferulic acid*** | 338+323 and 339+295 | 194 |

*: The reading was made for the compound, considering the contribution originated by adding ethanethiol (this is why two areas are read for each pair of ions).

1. **Solid analysis**

The total and volatile solids were analyzed by taking 2 g of raw material from a previously calcined crucible. Then, the sample was taken to the muffle at 105°C for 6 hours. Next, the sample was cooled in a desiccator and weighed (total solids determination). Afterward, the same moisture-free sample is taken to the muffle at 550°C for 2 hours. It is then cooled in the desiccator and weighed (determination of volatile solids) [7].

**References**

[1]. Sluiter A, Ruiz RO, Scarlata C, Sluiter J, Templeton D (2008) Determination of extractives in biomass. Technical Report NREL/TP-510–42619. Golden, Colorado

[2]. Luque de Castro MD, Priego-Capote F (2010) Soxhlet extraction: past and present panacea. J Chromatogr A 1217(16):2383–2389. https:// doi. org/ 10. 1016/J. CHROMA. 2009. 11. 027

[3]. Sluiter A, Ruiz RO, Scarlata C, Sluiter J, Templeton D (2005) Determination of ash in biomass. Technical Report NREL/TP-510–42622. Golden, Colorado

[4]. Han JS, Rowell JS (1996) Chemical composition of fibers. In: Paper and Composites from agrobased resources, 1st edn, vol. 1. CRC Press, Boca Raton, pp 83–134

[5]. Sluiter A, Ruiz RO, Scarlata C, Sluiter J, Templeton D (2012) Determination of structural carbohydrates and lignin in biomass. Technical Report NREL/TP-510–42618. Golden, Colorado

[6]. Majira A., Godon B., Foulon L., van der Putten J.C., Cézard L., Thierry M., Pion F., Bado-Nilles A., Pandard P., Jayabalan T., Aguié-Béghin V., Ducrot P.H., Lapierre C., Marlair G., Gosselink R.J.A., Baumberger S., Cottyn B. (2019). Enhancing the antioxidant activity of technical lignins by combining solvent fractionation and ionic liquid treatment. ChemSusChem. doi:10.1002/cssc.201901916

[7]. Sluiter A, Hames B, Hyman D, Payne C, Ruiz R, Scarlata C, Sluiter J, Templeton D, Wolfe J. (2008) Determination of Total Solids in Biomass and Total Dissolved Solids in Liquid Process Samples. Technical Report NREL/TP-510-42621. Golden, Colorado
