# Supplementary material for: Strategy for the analysis of lignocellulosic biomass to select a viable transformation route in the Colombian context
Source: Environ Sci Pollut Res Int. 2024 May 2;32(48):27741–62. doi: 10.1007/s11356-024-32975-x (PMC12696050; doi:10.1007/s11356-024-32975-x)
Supplement: Supplementary file 2 — Supplementary file2 (DOCX 48 KB) [file 11356_2024_32975_MOESM2_ESM.docx]

**Supplementary Material 2**

**Title:** Analysis of lignocellulosic biomass composition for selection of transformation routes in the Colombian context

**Authors:** Sara Piedrahita-Rodríguez^1^, Andrés-Felipe Alzate-Ramírez^1^, Stéphanie Baumberger^2^, Laurent Cézard^2^, Mariana

Ortiz-Sánchez^1^, Diego Alexander Escobar García^3^, Ana María Zetty Arenas^1^, Konstantinos Moustakas^4^, Carlos Ariel Cardona Alzate^1*^

^1^Instituto de Biotecnología y Agroindustria, Departamento de Ingeniería Química, Universidad Nacional de Colombia, Manizales, Caldas, Zip Code: 170003, Colombia

^2^Institut Jean-Pierre Bourgin (IJPB), INRAE, AgroParisTech, Université Paris-Saclay,78000 Versailles, France

^3^Universidad Nacional de Colombia, Sede Manizales, Facultad de Ingeniería y Arquitectura, Departamento de Ingeniería Civil, Grupo de Investigación en Movilidad Sostenible (GIMS), Campus La Nubia, Manizales, Caldas 170003, Colombia

^4^National Technical University of Athens, Unit of Environmental Science & Technology. School of Chemical Engineering, Greece

***Corresponding author:** ccardonaal@unal.edu.co

**SM2. Review for graphical composition model**

Lignocellulosic biomass mainly comprises cellulose, hemicellulose, lignin, extractives, ash, and sometimes traces of proteins, starch, or fats. These biomass fractions lead to different transformation technologies. In order to propose the graphical composition model, a review of the transformation processes that have been studied for each of the lignocellulosic biomass fractions was carried out to define the transformation limits.

- Cellulose, hemicellulose, and lignin

| **Raw material** | **Cellulose** | **Hemicellulose** | **Lignin** | **Ratio** (cel+hem/lig) | **Transformation route** | **Product(s)** | **Reference** |
| --- | --- | --- | --- | --- | --- | --- | --- |
| Sugarcane straw | 31.7 | 27.0 | 31.1 | 1.89 | Fermentation | Xylitol | [1] |
| Sugarcane bagasse | 41.95 | 21.7 | 24.11 | 2.64 | Fed-batch simultaneous saccharification and fermentation | Ethanol | [2] |
| Wheat bran | 32.43 | 26.34 | 20.15 | 2.92 | Fermentation | PHB | [3] |
| Cassava stem | 39.5 | 16.6 | 37.5 | 1.49 | Fermentation | Ethanol | [4] |
| Corncob | 41.18 | 33.37 | 18.68 | 3.99 | Fed-batch Fermentation | Xylitol | [5] |
| Rice straw | 36.1 | 27.0 | 13.7 | 4.60 | Integrated process (ABE fermentation) | Butanol | [6] |
| Rice straw | 49.2 | 29.2 | 16.3 | 4.81 | ABE fermentation | Acetone, Ethanol, and Butanol | [7] |
| Aloe peel waste | 37.80 | 28.30 | 14.10 | 4.69 | Anaerobic digestion with alkali pretreatment | Biogas | [8] |
| Corn stover | 42.43 | 33.80 | 5.43 | 9.6 | Fermentation with furfural tolerant microorganism | L-Lactic acid | [9] |
| Sorghum stover | 42.03 | 24.53 | 9.89 | 6.73 | Fermentation | Ethanol | [10] |
| Spruce | 42 | 5.7 | 28.2 | 1.69 | Enzymatic saccharification | Fermentable sugars | [11] |
| Aspen | 44 | 19 | 19 | 3.31 | Enzymatic saccharification | Fermentable sugars | [11] |
| Oil palm EFB | 37.26 | 14.62 | 31.68 | 1.64 | Fermentation | Ethanol | [12] |
| Oil palm EFB | 40.6 | 36.6 | 19.6 | 3.94 | Simultaneous saccharification and fermentation | Succinic acid | [13] |
| Oil palm EFB fibers | 28.3 | 36.6 | 35.1 | 1.85 | Enzymatic hydrolysis | Fermentable sugars | [14] |
| Wheat straw | 36.7 | 21.5 | 12.5 | 4.66 | Fermentation | Lipid production | [15] |
| Rice straw | 35.8 | 17.5 | 14.3 | 3.72 | Fermentation | Butanol | [16] |
| Maize stem | 20 | 52 | 30 | 2.4 | Enzymatic saccharification | Fermentable sugars | [17] |
| Sugarcane bagasse | 42.41 | 21.92 | 20.31 | 3.18 | Fed-batch fermentation | Butanol | [18] |
| Corn stover | 31 | 20.1 | 24.1 | 2.12 | Fermentation | Butanol | [19] |

PHB: Polyhydrolybutyrate; EFB: empty fruit bunches;

- Lignin

| **Raw material** | **Lignin** | **Transformation route** | **Product(s)** | **Reference** |
| --- | --- | --- | --- | --- |
| Spruce | 27.73 | Downstream processing of bio-oil | Hydroxyacetaldehyde, Acetic acid, Levoglucosan, Hydroxypropanone, 2(5H)-furanone, 5-(Hydroxymethyl)-2-furaldehyde, 2-Hydroxy-2-cyclopentene-1-one, 2-Furaldehyde, 4-Methyl guaiacol, Guaiacol, Syringol | [20] |
| Salix | 22.5 |  |  |  |
| Miscanthus | 21.4 |  |  |  |
| Wheat straw | 15.96 |  |  |  |
| Rice straw | 7.69 | Pyrolysis | Bio-oil and phenols compounds | [21] |
| Southwest birch | 23.4 | Auto-catalyzed organosolv pretreatment and enzymatic hydrolysis | High-quality lignin for further processing | [22] |

- Extractives

| **Raw material** | **Extractives** | **Transformation route** | **Product(s)** | **Reference** |
| --- | --- | --- | --- | --- |
| Chestnut wood shell | 26.87 (in hot water) | Methanol/water 80/20 | Phenols | [23] |
| Eucalyptus bark | 5.31 (in hot water) | Ethanol/water 50/50 | Phenols | [23] |
| Scots pine wood | 50 | Accelerated  solvent extraction | Fatty acids, Resin acids, Lignans, Sterols, Steryl esters, triglycerides | [24] |
| Orange peel | 185,493 (mg/g) TPC | Ultrasound-assisted extraction | Naringin and Herespidin | [25] |
| Iberian  white birch | 4.49 | Ultrasound-assisted extraction | Phenols | [26] |
| Sweet chestnut | 6.88 |  |  |  |
| Black locust | 4.49 |  |  |  |
| Myrtle pericarp | 250 (mg/g) TPC | Microwave-assisted extraction | Tannins, anthocyanins, flavonoids | [27] |
| *Phyllanthus amarus* Schum & Thonn | 52.97 (mg/g) TPC | Ultrasound-assisted extraction and pressurized liquid extraction | Gallic acid, tannins, flavonoids and lignans | [28] |
| Spruce bark | 997.61 (mg/g) TPC | Supercritical fluid extraction | Ferulic acid and p-coumaric acid | [29] |
| Piper betle | 289.05 (mg/g) TPC | Ultrasound-assisted extraction | Saponins, tannins, steroids | [30] |

- Volatile and total solids

| **Raw material** | **VS** | **TS** | **C/N ratio** | **BI** | **Transformation route** | **Product(s)** | **Reference** |
| --- | --- | --- | --- | --- | --- | --- | --- |
| Potato waste | 95 | 19 | 36 | 500 | Anaerobic digestion | Methane | [31] |
| Sugar beet leaf | 84 | 11 | 14 | 763.6 |  |  |  |
| Aloe peel waste | 8.33 | 12.49 | 34.52 | 66.69 | Anaerobic digestion with alkali pretreatment | Methane | [8] |
| Palm oil mill effluent | 3.3 | 4.2 | N.R. | 0.78 | Thermophilic fermentation and  mesophilic methanogen process | Biohythane | [32] |
| Sugarcane bagasse | 85.8 | 88.9 | N.R. | 0.96 | Anaerobic digestion and combined heat and power  (CHP) system | Biogas to electrical energy | [33] |
| Coffee husk | 86.1 | 93.0 | N.R. | 0.93 |  |  |  |
| Bean straw | 84.4 | 92.3 | N.R. | 0.91 |  |  |  |
| Rice straw | 70.5 | 90.6 | N.R. | 0.78 |  |  |  |
| Banana leaves | 82.3 | 90.9 | N.R. | 0.91 |  |  |  |
| Cane straw | 86.8 | 90.5 | N.R. | 0.96 |  |  |  |
| Corn straw | 85.1 | 90.6 | N.R. | 0.94 |  |  |  |
| Buckwheat hull | 98.1 | 92.3 | 43.8 | 1.06 | Anaerobic digestion with different pretreatment | Methane | [34] |
| Pineapple peels | N.R. | 50.84 | 2.76 | N.D. | Anaerobic digestion | Methane | [35] |
| Spinach waste | N.R. | 64.90 | 1.14 | N.D. |  |  |  |
| Sugarcane bagasse | N.R. | 66.79 | 5.86 | N.D. |  |  |  |
| Banana peels | N.R. | 59.49 | 3.49 | N.D. |  |  |  |
| Papaya peels | N.R. | 53.04 | 1.72 | N.D. |  |  |  |

BI: Biodegradability index (VS/TS); TPC: Total phenolic compounds; N.R.: Not Reported; N.D. Not Determined.

- Protein, pectin, lipids, starch and others

| **Raw material** | **Component** | **Transformation route** | **Product(s)** | **Reference** |
| --- | --- | --- | --- | --- |
| Apple pomace | 11.7 | Enzymatic hydrolysis, precipitation and acid hydrolysis | Dietary fiber | [36] |
| Pear pomace | 13.4 |  |  |  |
| Avocado seed | Lipids: 1.87 Protein: 1.95 | Solvent extraction | Fatty acids | [37] |
| Corn | Starch: 40.40 | Enzymatic saccharification | Starch | [38] |
| Cassava | Starch: 71.05 | Enzymatic saccharification | Starch | [38] |
| Brewer’s spent grain | Starch: 0.32 | Conventional hydrolysis | Fermentable sugars | [39] |
| Paper waste | Starch: 20 | Hydrothermal reaction with Phosphomolybdic acid | Glycolic acid | [40] |
| Corn stover | Starch: 3.82 | Enzymatic saccharification | Amylose | [41] |

Energetic:

- Moisture, Volatile matter, fixed carbon, ash, HHV.

| **Raw material** | **Moisture** | **Volatile matter** | **Fixed carbon** | **Ash** | **HHV (MJ/kg)** | **Transformation route** | **Product(s)** | **Reference** |
| --- | --- | --- | --- | --- | --- | --- | --- | --- |
| Wheat straw | 4.2 | 73.2 | 18.2 | 4.39 | N.R. | Pyrolysis | Pyrolysis products (solid, liquid and gas) | [42] |
| Rape straw | 3.7 | 76.5 | 16.1 | 3.69 | N.R. | Pyrolysis | Pyrolysis products (solid, liquid and gas) | [42] |
| Spruce + bark | 6.9 | 68.9 | 19.3 | 4.88 | N.R. | Pyrolysis | Pyrolysis products (solid, liquid and gas) | [42] |
| Cardoon steams | N.R. | 77.7 | 14.7 | 7.6 | 21.5 | Pyrolysis | Biochar and gas | [43] |
| Cardoon leaves | N.R. | 59.5 | 10.9 | 29.6 | 17.9 | Pyrolysis | Biochar and gas | [43] |
|  | 5.98 | 89.85 | N.R. | 2.89 | 27.39 | Methanolysis | Bio-oil | [44] |
| Forest residues | Elemental composition: 50.9 C, 6.2 H, 0.02 S, 41.8 O. | | | | 20.5 | Gasification | Syngas and methanol | [45] |

N.R.: Not Reported

- **References:**

[1]. Hernández-Pérez, A. F., de Arruda, P. V., & Felipe, M. das G. de A. (2016). Sugarcane straw as a feedstock for xylitol production by Candida guilliermondii FTI 20037. Brazilian Journal of Microbiology, 47(2), 489–496. doi:10.1016/j.bjm.2016.01.019

[2]. Gao, Y., Xu, J., Yuan, Z., Jiang, J., Zhang, Z., & Li, C. (2018). Ethanol production from sugarcane bagasse by fed-batch simultaneous saccharification and fermentation at high solids loading. Energy Science & Engineering, 6(6), 810–818. doi:10.1002/ese3.257

[3]. Annamalai, N., & Sivakumar, N. (2016). Production of polyhydroxybutyrate from wheat bran hydrolysate using Ralstonia eutropha through microbial fermentation. Journal of Biotechnology, 237, 13–17. doi:10.1016/j.jbiotec.2016.09.001

[4]. Tanaka, K., Koyama, M., Pham, P. T., Rollon, A. P., Habaki, H., Egashira, R., & Nakasaki, K. (2019). Production of high-concentration bioethanol from cassava stem by repeated hydrolysis and intermittent yeast inoculation. International Biodeterioration & Biodegradation, 138, 1–7. doi:10.1016/j.ibiod.2018.12.007

[5]. Ping, Y., Ling, H.-Z., Song, G., & Ge, J.-P. (2013). Xylitol production from non-detoxified corncob hemicellulose acid hydrolysate by Candida tropicalis. Biochemical Engineering Journal, 75, 86–91. doi:10.1016/j.bej.2013.03.022

[6]. Wu, J., Dong, L., Liu, B., Xing, D., Zhou, C., Wang, Q., Wu X., Feng L., Cao, G. (2020). A novel integrated process to convert cellulose and hemicellulose in rice straw to biobutanol. Environmental Research, 109580. doi:10.1016/j.envres.2020.109580

[7]. Amiri, H., Karimi, K., & Zilouei, H. (2014). Organosolv pretreatment of rice straw for efficient acetone, butanol, and ethanol production. Bioresource Technology, 152, 450–456. doi:10.1016/j.biortech.2013.11.038

[8]. Huang, X., Yun, S., Zhu, J., Du, T., Zhang, C., & Li, X. (2016). Mesophilic anaerobic co-digestion of aloe peel waste with dairy manure in the batch digester: Focusing on mixing ratios and digestate stability. Bioresource Technology, 218, 62–68. doi:10.1016/j.biortech.2016.06.070

[9]. Klongklaew, A.; Unban, K.; Kalaimurugan, D.; Kanpiengjai, A.; Azaizeh, H.; Schroedter, L.; Schneider, R.; Venus, J.; Khanongnuch, C. (2023). Bioconversion of Dilute Acid Pretreated Corn Stover to L-Lactic Acid Using Co-Culture of Furfural Tolerant Enterococcus mundtii WX1 and Lactobacillus rhamnosus SCJ9. Fermentation, 9, 112. https://doi.org/10.3390/fermentation9020112

[10]. Sathesh-Prabu, C., & Murugesan, A. G. (2011). Potential utilization of sorghum field waste for fuel ethanol production employing Pachysolen tannophilus and Saccharomyces cerevisiae. Bioresource Technology, 102(3), 2788–2792. doi:10.1016/j.biortech.2010.11.097

[11]. Wang, Z., Winestrand, S., Gillgren, T., & Jönsson, L. J. (2018). Chemical and structural factors influencing enzymatic saccharification of wood from aspen, birch and spruce. Biomass and Bioenergy, 109, 125–134. doi:10.1016/j.biombioe.2017.12.020

[12]. Sudiyani, Y., Styarini, D., Triwahyuni, E., Sudiyarmanto, Sembiring, K. C., Aristiawan, Y., … Han, M. H. (2013). Utilization of Biomass Waste Empty Fruit Bunch Fiber of Palm Oil for Bioethanol Production Using Pilot–Scale Unit. Energy Procedia, 32, 31–38. doi:10.1016/j.egypro.2013.05.005

[13]. Akhtar, J., & Idris, A. (2017). Oil palm empty fruit bunches a promising substrate for succinic acid production via simultaneous saccharification and fermentation. Renewable Energy, 114, 917–923. doi:10.1016/j.renene.2017.07.113

[14]. Palamae, S., Dechatiwongse, P., Choorit, W., Chisti, Y., & Prasertsan, P. (2017). Cellulose and hemicellulose recovery from oil palm empty fruit bunch (EFB) fibers and production of sugars from the fibers. Carbohydrate Polymers, 155, 491–497. doi:10.1016/j.carbpol.2016.09.004

[15]. Yu, X., Zheng, Y., Dorgan, K. M., & Chen, S. (2011). Oil production by oleaginous yeasts using the hydrolysate from pretreatment of wheat straw with dilute sulfuric acid. Bioresource Technology, 102(10), 6134–6140. doi:10.1016/j.biortech.2011.02.081

[16]. Valles, A., Capilla, M., Álvarez-Hornos, F. J., García-Puchol, M., San-Valero, P., & Gabaldón, C. (2021). Optimization of alkali pretreatment to enhance rice straw conversion to butanol. Biomass and Bioenergy, 150, 106131. doi:10.1016/j.biombioe.2021.106131

[17]. Zhu, Z., Liu, Y., Gómez, L. D., Wei, T., Yang, X., Simister, R., … Macquarrie, D. J. (2021). Thermochemical pretreatments of maize stem for sugar recovery: Comparative evaluation of microwave and conventional heating. Industrial Crops and Products, 160, 113106. doi:10.1016/j.indcrop.2020.113106

[18]. Pang, Z.-W., Lu, W., Zhang, H., Liang, Z.-W., Liang, J.-J., Du, L.-W., … Feng, J.-X. (2016). Butanol production employing fed-batch fermentation by Clostridium acetobutylicum GX01 using alkali-pretreated sugarcane bagasse hydrolysed by enzymes from Thermoascus aurantiacus QS 7-2-4. Bioresource Technology, 212, 82–91. doi:10.1016/j.biortech.2016.04.013

[19]. Xu, G.-C., Ding, J.-C., Han, R.-Z., Dong, J.-J., & Ni, Y. (2016). Enhancing cellulose accessibility of corn stover by deep eutectic solvent pretreatment for butanol fermentation. Bioresource Technology, 203, 364–369. doi:10.1016/j.biortech.2015.11.002

[20]. Butler, E., Devlin, G., Meier, D., & McDonnell, K. (2013). Characterisation of spruce, salix, miscanthus and wheat straw for pyrolysis applications. Bioresource Technology, 131, 202–209. doi:10.1016/j.biortech.2012.12.013

[21]. Liang, J., Lin, Y., Wu, S., Liu, C., Lei, M., & Zeng, C. (2015). Enhancing the quality of bio-oil and selectivity of phenols compounds from pyrolysis of anaerobic digested rice straw. Bioresource Technology, 181, 220–223. doi:10.1016/j.biortech.2015.01.056

[22]. Wen, J.-L., Xue, B.-L., Sun, S.-L., & Sun, R.-C. (2013). Quantitative structural characterization and thermal properties of birch lignins after auto-catalyzed organosolv pretreatment and enzymatic hydrolysis. Journal of Chemical Technology & Biotechnology, 88(9), 1663–1671. doi:10.1002/jctb.4017

[23]. Vázquez, G., Fontenla, E., Santos, J., Freire, M. S., González-Álvarez, J., & Antorrena, G. (2008). Antioxidant activity and phenolic content of chestnut (Castanea sativa) shell and eucalyptus (Eucalyptus globulus) bark extracts. Industrial Crops and Products, 28(3), 279–285. doi:10.1016/j.indcrop.2008.03.003

[24]. Verkasalo, E.; Roitto, M.; Möttönen, V.; Tanner, J.; Kumar, A.; Kilpeläinen, P.; Sikanen, L.; Ilvesniemi, H. Extractives of Tree Biomass of Scots Pine (Pinus sylvestris L.) for Biorefining in Four Climatic Regions in Finland. (2022). Lipophilic Compounds, Stilbenes, and Lignans. Forests, 13, 779. https://doi.org/10.3390/f13050779

[25]. Khan, M. K., Abert-Vian, M., Fabiano-Tixier, A.-S., Dangles, O., & Chemat, F. (2010). Ultrasound-assisted extraction of polyphenols (flavanone glycosides) from orange (Citrus sinensis L.) peel. Food Chemistry, 119(2), 851–858. doi:10.1016/j.foodchem.2009.08.046

[26]. Sillero L., Morales A., Fernández-Marín R., Hernández-Ramos F., Davila I., Erdocia X., Labidi J. (2021). Study of different extraction methods of bioactive molecules from different tree species. Chemical Engineering Transactions. 86. 31-36

[27]. Bouaoudia-Madi, N., Boulekbache-Makhlouf, L., Kadri, N., Dahmoune, F., Remini, H., Dairi, S., … Madani, K. (2017). Phytochemical analysis of Myrtus communis plant: Conventional versus microwave assisted-extraction procedures. Journal of Complementary and Integrative Medicine, 14(4). doi:10.1515/jcim-2016-0098

[28]. Sousa, A. D., Maia, A. I. V., Rodrigues, T. H. S., Canuto, K. M., Ribeiro, P. R. V., de Cassia Alves Pereira, R., … de Brito, E. S. (2016). Ultrasound-assisted and pressurized liquid extraction of phenolic compounds from Phyllanthus amarus and its composition evaluation by UPLC-QTOF. Industrial Crops and Products, 79, 91–103. doi:10.1016/j.indcrop.2015.10.045

[29]. Talmaciu, A. I., Ravber, M., Volf, I., Knez, Ž., & Popa, V. I. (2016). Isolation of bioactive compounds from spruce bark waste using sub- and supercritical fluids. The Journal of Supercritical Fluids, 117, 243–251. doi:10.1016/j.supflu.2016.07.001

[30]. Ali, A., Lim, X. Y., Chong, C. H., Mah, S. H., & Chua, B. L. (2018). Optimization of ultrasound-assisted extraction of natural antioxidants from Piper betle using response surface methodology. LWT, 89, 681–688. doi:10.1016/j.lwt.2017.11.033

[31]. Parawira, W., Murto, M., Zvauya, R., & Mattiasson, B. (2004). Anaerobic batch digestion of solid potato waste alone and in combination with sugar beet leaves. Renewable Energy, 29(11), 1811–1823. doi:10.1016/j.renene.2004.02.005

[32]. Mamimin, C., Singkhala, A., Kongjan, P., Suraraksa, B., Prasertsan, P., Imai, T., & O-Thong, S. (2015). Two-stage thermophilic fermentation and mesophilic methanogen process for biohythane production from palm oil mill effluent. International Journal of Hydrogen Energy, 40(19), 6319–6328. doi:10.1016/j.ijhydene.2015.03.068

[33]. López-Dávila, E., Jiménez Hernández, J., López González, L. M., Barrera Cardoso, E, L., Bravo Amarante, E., Contreras Velázquez, L, M., & Romero-Romero, O. (2022). Biochemical methane potential of agro-wastes as a renewable source alternative for electrical energy production in Cuba. Ciencia y Tecnología Agropecuaria, 23(1), e1890. https://doi.org/10.21930/rcta.vol23_num1_art:1890

[34]. Mirko, C., Daniela, P., Chiara, T., & Giovanni, G. (2021). Pretreatments for enhanced biomethane production from buckwheat hull: Effects on organic matter degradation and process sustainability. Journal of Environmental Management, 285, 112098. doi:10.1016/j.jenvman.2021.112098

[35]. Anitha, M., Kamarudin, S. K., Shamsul, N. S., & Kofli, N. T. (2015). Determination of bio-methanol as intermediate product of anaerobic co-digestion in animal and agriculture wastes. International Journal of Hydrogen Energy, 40(35), 11791–11799. doi:10.1016/j.ijhydene.2015.06.072

[36]. Nawirska, A., & Kwaśniewska, M. (2005). Dietary fibre fractions from fruit and vegetable processing waste. Food Chemistry, 91(2), 221–225. doi:10.1016/j.foodchem.2003.10.005

[37]. Bora PS, Narain N, Rocha RV, Queiroz Paulo M. (2001). Characterization of the oils from the pulp and seeds of avocado (cultivar: Fuerte) fruits. Grasasaceites CSIC 52(3-4):171-4.

[38]. Quintero, J.A., Dávila, J.A., Moncada, J., Giraldo, O.H., Cardona, C.A. (2016). Analysis, and characterization of starchy and cellulosic materials after enzymatic modification. DYNA 83(197), pp. 44-51.

[39]. Luft, L., Confortin, T. C., Todero, I., Ugalde, G., Zabot, G. L., & Mazutti, M. A. (2018). Transformation of residual starch from brewer’s spent grain into fermentable sugars using supercritical technology. The Journal of Supercritical Fluids, 140, 85–90. doi:10.1016/j.supflu.2018.06.006

[40]. Qiao Y., Wang X., Dai X. (2021). Experimental and kinetic study of the conversion of waste starch into glycolic acid over phosphomolybdic acid RSC Adv. 11(49): 30961–30970. doi:10.1039/d1ra05890h

[41]. You, C., Chen, H., Myung, S., Sathitsuksanoh, N., Ma, H., Zhang, X.-Z., … Zhang, Y.-H. P. (2013). Enzymatic transformation of nonfood biomass to starch. Proceedings of the National Academy of Sciences, 110(18), 7182–7187. doi:10.1073/pnas.1302420110

[42]. Burhenne, L., Messmer, J., Aicher, T., & Laborie, M.-P. (2013). The effect of the biomass components lignin, cellulose and hemicellulose on TGA and fixed bed pyrolysis. Journal of Analytical and Applied Pyrolysis, 101, 177–184. doi:10.1016/j.jaap.2013.01.012

[43]. Damartzis, T., Vamvuka, D., Sfakiotakis, S., & Zabaniotou, A. (2011). Thermal degradation studies and kinetic modeling of cardoon (Cynara cardunculus) pyrolysis using thermogravimetric analysis (TGA). Bioresource Technology, 102(10), 6230–6238. doi:10.1016/j.biortech.2011.02.060

[44]. Carvalho, L., Furusjö, E., Kirtania, K., Wetterlund, E., Lundgren, J., Anheden, M., & Wolf, J. (2017). Techno-economic assessment of catalytic gasification of biomass powders for methanol production. Bioresource Technology, 237, 167–177. doi:10.1016/j.biortech.2017.02.019

[45]. Li, Z.-K., Cheng, J.-Y., Yan, H.-L., Yan, J.-C., Lei, Z.-P., Ren, S.-B., … Shui, H.-F. (2021). Pretreatment of sweet sorghum stalk with aqueous hydrogen peroxide for enhancing methanolysis and property of the bio-oil. Renewable Energy, 175, 1127–1136. doi:10.1016/j.renene.2021.05.052
