# Supplementary material for: Strategy for the analysis of lignocellulosic biomass to select a viable transformation route in the Colombian context
Source: Environ Sci Pollut Res Int. 2024 May 2;32(48):27741–62. doi: 10.1007/s11356-024-32975-x (PMC12696050; doi:10.1007/s11356-024-32975-x)
Supplement: Supplementary file 3 — Supplementary file3 (DOCX 296 KB) [file 11356_2024_32975_MOESM3_ESM.docx]

**Supplementary Material 3**

**Title:** Analysis of lignocellulosic biomass composition for selection of transformation routes in the Colombian context

**Authors:** Sara Piedrahita-Rodríguez^1^, Andrés-Felipe Alzate-Ramírez^1^, Stéphanie Baumberger^2^, Laurent Cézard^2^, Mariana Ortiz-Sánchez^1^, Diego Alexander Escobar García^3^, Ana María Zetty Arenas^1^, Konstantinos Moustakas^4^, Carlos Ariel Cardona Alzate^1*^

^1^Instituto de Biotecnología y Agroindustria, Departamento de Ingeniería Química, Universidad Nacional de Colombia, Manizales, Caldas, Zip Code: 170003, Colombia

^2^Institut Jean-Pierre Bourgin (IJPB), INRAE, AgroParisTech, Université Paris-Saclay,78000 Versailles, France

^3^Universidad Nacional de Colombia, Sede Manizales, Facultad de Ingeniería y Arquitectura, Departamento de Ingeniería Civil, Grupo de Investigación en Movilidad Sostenible (GIMS), Campus La Nubia, Manizales, Caldas 170003, Colombia

^4^National Technical University of Athens, Unit of Environmental Science & Technology. School of Chemical Engineering, Greece

***Corresponding author:** ccardonaal@unal.edu.co

**SM3. Environmental Assessment: System boundaries and LCA inventories.**

1. **Results**

**Figure 1** and **Figure 2** show the System boundary considered for the biorefinery schemes. The **Table 1** and **Table 2** show the inventory of the agronomic stage for the raw materials. For the case of PP (**Figure 1**) the system boundaries were divided into three systems: (i) seedlings Production; (ii) pinus patula cultivation; and (iii) PP processing. A description of the systems for these schemes was taken from Garcia et al (2017).

- 1. **Seedlings production**

This system comprises all nursery activities divided into three subsystems. The first subsystem corresponded to the substrate preparation, which must be carefully chosen to guarantee seed germination. The seedbeds are made of wood, guadua, and cement; sand and soil and used as substrates. Subsequently, fungicide (thiabendazole) is added at a rate of 0.125 cm^3^/seed. Then, phenol formaldehyde and water at a rate of 0.2 kg/seed are added to improve the seeds germination capacity. Finally, an herbicide (molluscicide, 0.083 g/seed is added to control the proliferation of slugs and snails. Subsystem two consisted of seedling preparation. It starts with manually transplanting the seedlings into high-density polystyrene bags before planting them into the soil. Subsequently, an insecticide (cypermethrin) is added at a 2.2 µg/seed rate. Subsystem 3 includes the application of agrochemicals, starting with two fertilization processes with diammonium phosphate, borolik, and KCl, as recommended by Garcia et al. (2017).

- 1. ***Pinus patula* cultivation**

This system includes several subsystems. The first, called site preparation, involves removing impurities, weeds, and other invasive plants from the land. This activity is carried out with a tractor connected to a roller packer. Subsequently, an herbicide (glyphosate, 1.5 L/ha) is applied with the help of a sprayer. The land is then plowed to remove the soil and reduce its hardness (a process carried out with a tractor). The next subsystem consists of planting with a seed drill. The third subsystem is harvesting and silviculture, which involves three harvests and a cutting cycle. Each cycle has a rotation period (there are three in total: the first and third of 7 years and the second of 5 years). In each cutting cycle, silviculture and harvesting activities are repeated. For this, fertilization is performed using NPK (15%-38%-10%) at 55.55 g/ha, sodium borate at 8.88 g/ha, and sulfate zinc at 22.22 g/ha. The best time to carry out this activity is approximately after the first thinning (in the sixth year). Consequently, the fertilization stage occurs in the second and third cycles. Subsequently, pruning is carried out to increase the growth of *pinus patula* (a pruning saw is used). Finally, the pine is harvested with chainsaws, and the wood is accumulated in storage centers with the help of a tractor connected to a Koller.

- 1. **PP processing**

This system involves the processing of *pinus patula* according to the proposed biorefineries of PP scheme. The inputs and outputs of the schemes are included in the diagram.


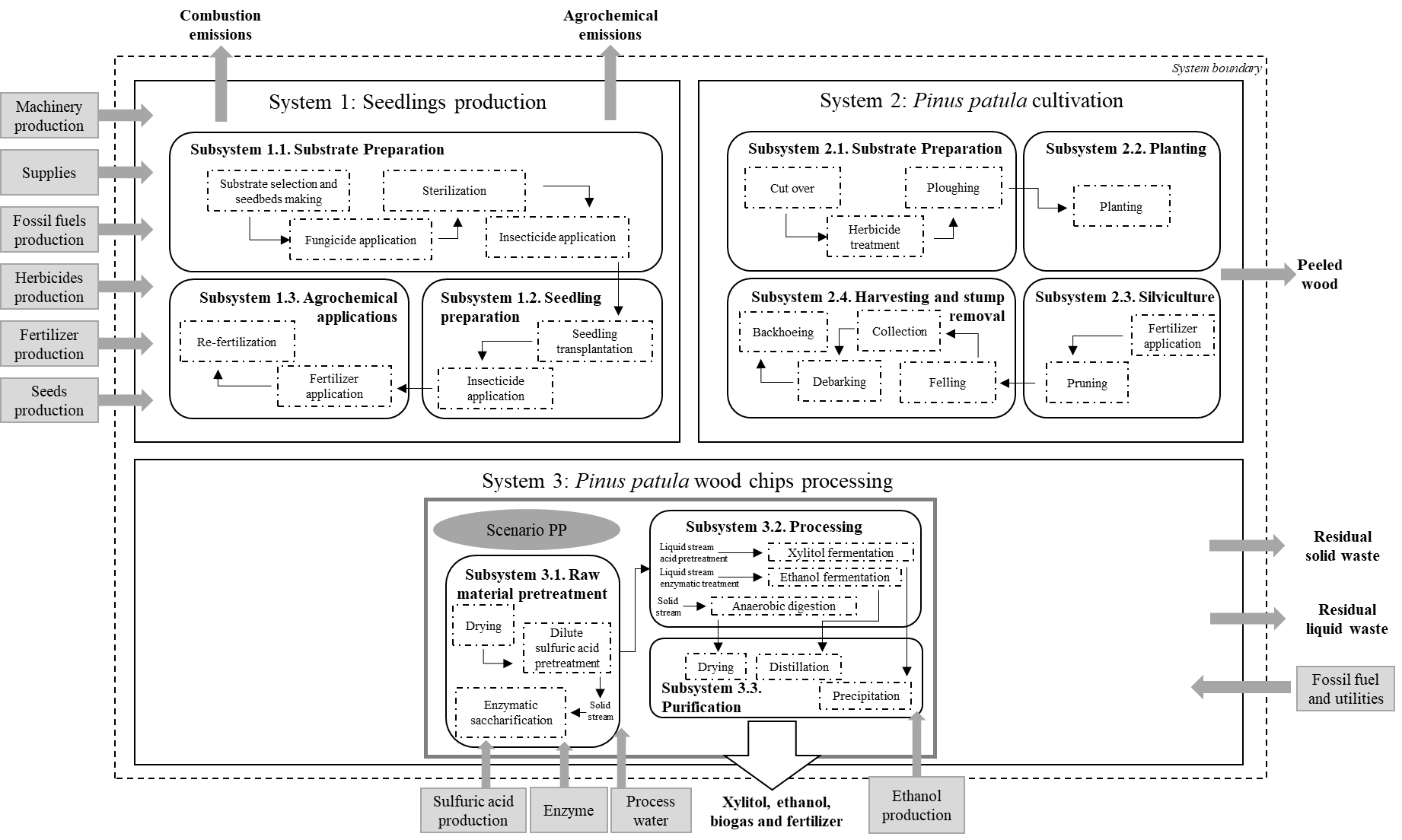


**Figure 1. System boundary for PP scheme**

For the bagasse from NCS production case (**Figure 2**), three systems were defined: (i) sugarcane from the NCS production crop; (ii) panela production; and (iii) bagasse from NCS production processing. The stages are described below.

- 1. **Sugarcane from NCS production crop**

The agricultural practices considered were land preparation, vegetative stage, and harvest. The material removal in the land preparation is the only activity carried out with machinery. The first fertilization is made at this stage. In the vegetative stage, the second fertilization and material removal are carried out. The harvest is conducted manually. The capture of CO_2_ by part of the total carbon stored in the crop was considered. In this sense, the carbon stored in the aerial biomass of the sugarcane crop was considered. The allometric equation for calculating aerial biomass reported by de Carvalho et al. (2019) was used. Carbon storage by biomass was calculated using the constant of 0.5 defined by the Intergovernmental Panel on Climate Change (IPCC 2016). The carbon sequestration rate for sugarcane was estimated by dividing carbon storage in biomass by the average age of the crop. Finally, CO_2_ uptake was calculated using the constant 3.67. The water footprint of the non-centrifuged sugarcane crop was estimated from the water requirements using the free software COPWAT 8.0. For this, climatic information was used the minimum temperature, maximum temperature, humidity, wind, sun, rain, and specific crop variables. The crop water requirements were calculated from the evapotranspiration and the effective precipitation according to the methodology reported by Hoekstra et al., (2009). This information was used to calculate the green and blue water footprints. Finally, the gray water footprint was calculated as reported by Novoa et al., (2019), considering a leaching rate of 10%, the maximum allowed concentration of nitrogen in the water according to Colombian regulations (0.01g/L), the concentration of natural nitrogen in bodies of water and the amount of nitrogen added to the crop per hectare.

- 1. **Panela Production**

In Colombia, the panela production is manually conducted. The subsystems considered were grinding, filtration, cleaning, and concentration. The sugarcane juice extraction is carried out from a mill in the milling. Diesel is used as fuel in the sugarcane milling. 4.4 kg of bagasse from NCS production is generated per 10 kg of panela sugarcane. 0.056 diesel gallons are needed to process 10 kg of panela sugarcane. In the filtration and cleaning stage, a natural clarifier (e.g., tree sap) is added. In this stage, a residual stream so-called sludge is generated. For 6.6 kg of sugarcane juice, 0.014 kg of sludge are generated. In the concentration of sugarcane juice, antifoam is added. Small producers add vegetable oil as a defoaming agent. For every 6.6 kg of cane juice, 2 mL of vegetable oil are added. The concentration of the sugarcane juice is carried out in traditional stoves. Heat is produced by bagasse combustion. Wood is also used for heating when bagasse combustion is not sufficient for the process. In general, all the bagasse from NCS production generated in the previous grinding is used. In this research, complete combustion of the bagasse was considered. The residual currents generated in the concentration stage are steam, ashes, and impurities. Ashes are used as an additive in soil.

- 1. **Bagasse from NCS production processing**

This system involves the processing of the bagasse from NCS production according to the proposed biorefinery of bagasse from NCS production scheme. The inputs and outputs of the scheme were included in the diagram (**Figure 2**).


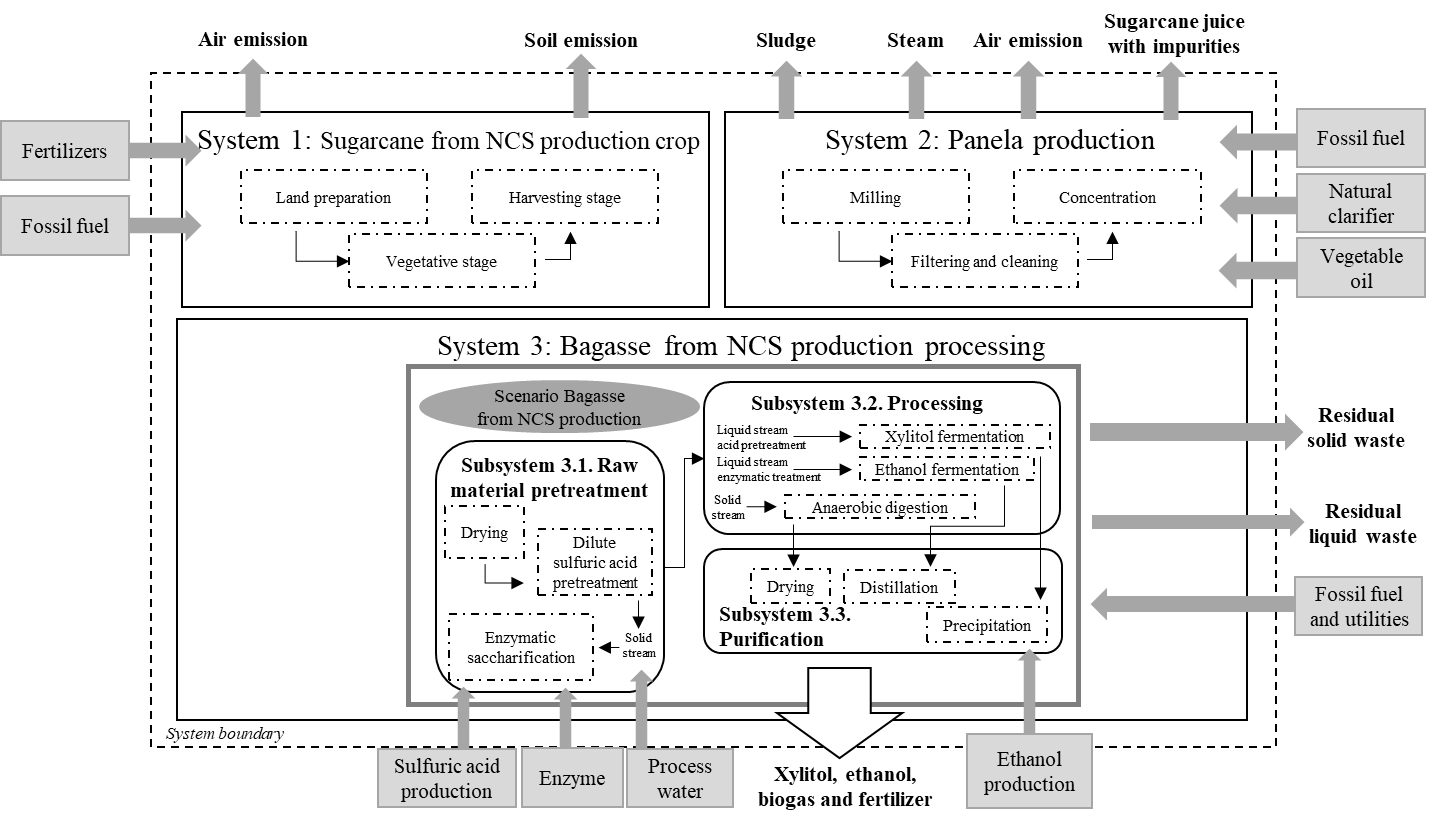


**Figure 2. System boundary for bagasse from NCS production scheme.**

**Table 1. *Pinus patula* production inventory for PP scheme.**

| 190 Pine trees (1 ha) | | | | | | | | | |
| --- | --- | --- | --- | --- | --- | --- | --- | --- | --- |
| System | Subsystem | Activity | Inputs | | | Outputs | | | Machinery |
|  |  |  | Item | Value | Unit | Item | Value | Units |  |
| Seeding production | Substrate preparation | Preparation | Wood | 158.27 | kg/ha | - | - | - | Manual |
|  |  |  | Cement | 31.73 | kg/ha | - | - | - | Manual |
|  |  | Fungicide application | Tiabendazole | 0.03825 | kg/ha | - | - | - | Manual |
|  |  | Sterilization | Phenol formaldehyde | 38.76 | kg/ha | - | - | - | Manual |
|  |  |  | Water | 24.7 | L/ha | - | - | - | Manual |
|  |  | Herbicide application | Molluscicide | 0.01583 | kg/ha | - | - | - | Manual |
|  | Seeding preparation | Seeding transplantation | High density polystyrene | 15.713 | kg/ha | - | - | - | Manual |
|  |  | Insecticide application | Cypermethrym | 0.00038 | kg/ha | - | - | - | Manual |
|  | Agrochemical applications | Fertilizer application | Diammonium phosphate DAP (18%-46%-0%) | 0.00262 | kg/ha | - | - | - | Manual |
|  |  | Refertilization | Diammonium phosphate DAP (18%-46%-0%) | 0.00262 | kg/ha | - | - | - | Manual |
| Pinus patula cultivation | Site preparation | Cut over | - | - | - | Fuel consumption | 22 | L/ha/y | Tractor and roller packer |
|  |  | Herbicide treatment | Glyphosate | 1.5 | L/ha |  |  |  | Sprayer |
|  |  | Ploughing | - | - | - | Fuel consumption | 48 | L/ha/y | Tractor and Plough |
|  | Planting | Planting | - | - | - |  |  |  | Seed Drill |
|  | Silviculture | Fertilizer application | NPK (15%-38%-10%) | 0.05500 | kg/ha | - | - | - | Knapsack sprayer |
|  |  |  | Sodium borate | 0.00880 | kg/ha |  |  |  |  |
|  |  |  | Zinc sulfate | 0.02200 | kg/ha |  |  |  |  |
|  |  | Pruning | - | - | - | Fuel consumption | 47 | L/ha/y | Pruning saw |
|  | Harvesting | Felling | - | - | - | Fuel consumption | 70,72 | L/ha/y | Chainsaw |
|  |  | Collection | - | - | - | Fuel consumption | 89,9 | L/ha/y | Tractor and Koller Tower |
|  |  | Debarking | - | - | - | Fuel consumption | 15,48 | L/ha/y | Debarker |
|  | Stump removal | Backhoeing | - | - | - | Fuel consumption | 12,56 | L/ha/y | Backhoe |
| Air emission | All the activities | | - | - | - | NO2 | 0.17 | µg | - |
|  |  |  | - | - | - | N2 | 0.67 | µg | - |
|  |  |  | - | - | - | NOx | 0.57 | µg | - |
|  |  |  | - | - | - | NH3 | 0.57 | µg | - |
|  |  |  | - | - | - | SO2 | 0.12 | g | - |
|  |  |  | - | - | - | CO2 | 39.3 | g | - |
|  |  |  | - | - | - | CO | 0.08 | g | - |
|  |  |  | - | - | - | VOC | 0.04 | g | - |
|  |  |  | - | - | - | N2O | 8 | µg | - |
|  |  |  | - | - | - | Pentane | 4 | µg | - |
|  |  |  | - | - | - | NMVOC | 0.4 | µg | - |
|  |  |  | - | - | - | CH4 | 1.6 | µg | - |
|  |  |  | - | - | - | Particulates | 14.5 | µg | - |

**Table 2. Panela production inventory for bagasse from NCS production scheme.**

| 1 kg panela | | | | | | | | | |
| --- | --- | --- | --- | --- | --- | --- | --- | --- | --- |
| System | Subsystem | Activity | Inputs | | | Outputs | | | Machinery |
|  |  |  | Item | Value | Units | Item | Value | Units |  |
| Agro-activities | Soil preparation | Ollado | - | - | - | - | - | - | Manual |
|  |  | Fertilizer addition | Organic fertilizer | 1 | kg/ha | 0 |  |  | Manual |
|  | Sowing | Fertilizer addition | Ash | 1 | kg/ha | 0 |  |  | Manual |
|  |  | Material removal | - | - | - | - | - | - | Manual |
|  | Harvesting | Cane cutting | Non-centrifuged cane | 300 | kg/ha | Non-centrifuged cane | 300 | kg/ha | Manual |
| Agro-industrial activities | Milling | Extraction of sugarcane juice | Juice | 90 | kg/ha | Non-centrifuged sugarcane bagasse | 60 | kg/ha | Crusher |
|  | Filtration and clarification | Balsa and cadillo emulsifying addities | Balso | 1 | kg/ha | Cachaza | 1 | kg/ha | Oven 1 |
|  |  |  | Cadillo | 1 | kg/ha | Biomass | 0.5 | kg/ha | Oven 2 |
|  |  |  | Water | 20 | kg/ha |  |  |  |  |
|  | Concentration and blending | Evaporation of water and solids concentration | Clarified juice | 108.5 | kg/ha | Juice | 59.5 | kg/ha | Oven 3 |
|  |  |  |  |  |  | Water vapor | 48.83 | kg/ha |  |
|  |  |  | Wood | 60 | kg/ha | Ash | 24 | kg/ha | Combustion furnace |
|  |  |  | Non-centrifuged sugarcane bagasse | 60 | kg/ha |  |  |  |  |
|  | Packaging | Molding, cooling and packaging | Honey | 59.7 | kg/ha | Panela | 39 | kg/ha | Oven 4 |
|  |  |  |  |  |  | Vapor | 24.69 | kg/ha |  |

1. **Reference**

García CA, Morales M, Quintero J, Aroca G, Cardona CA (2017) Environmental assessment of hydrogen production based on Pinus patula plantations in Colombia. Energy 139:606-616. https://doi.org/10.1016/j.energy.2017.08.012
